# Expected and Unexpected Reactivities of Homoleptic LiNacNac and Heteroleptic NacNacMg(TMP) β-Diketiminates toward Various Small Unsaturated Organic Molecules

**DOI:** 10.1021/acs.inorgchem.1c00549

**Published:** 2021-04-08

**Authors:** Richard M. Gauld, Jennifer R. Lynch, Alan R. Kennedy, Jim Barker, Jacqueline Reid, Robert E. Mulvey

**Affiliations:** † WestCHEM, Department of Pure and Applied Chemistry, University of Strathclyde, Glasgow G1 1XL, U.K.; ‡ Innospec Ltd., Innospec Manufacturing Park, Oil Sites Road, Ellesmere Port, Cheshire CH65 4EY, U.K.

## Abstract

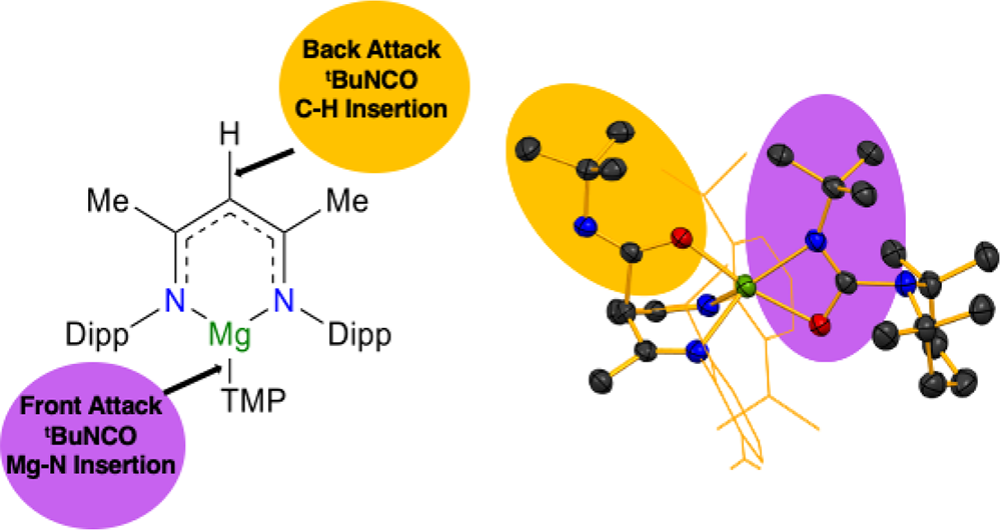

Homoleptic LiNacNac forms simple donor–acceptor complexes with *N*,*N*′-dicyclohexylcarbodiimide (CyN=C=NCy), triphenylphosphine oxide (Ph_3_P=O), and benzophenone (Ph_2_CO). These crystallographically characterized compounds could be regarded as model intermediates en route to reducing the N=C, P=O, and C=O bonds of unsaturated substrates. Heteroleptic NacNacMg(TMP) intriguingly functions as a TMP nucleophile both with *t*-BuNCO and *t*-BuNCS, producing a urea or thiourea derivative respectively attached to Mg, though the NacNac ligand in the former reaction also engages noninnocently with a second *t*-BuNCO molecule via insertion at the reactive NacNac backbone γ-carbon site.

## Abstract 2

Twofold insertion of *t*-BuNCO into NacNacMg(TMP) results in a modified NacNac ligand via attack at the “back” nucleophilic γ-carbon, potentially opening the door to new functionalized NacNac species, while in the “front” attack the base TMP surprisingly behaves as a nucleophile. This work also reveals crystallographically determined exemplary structures of NacNacLi coordinated to a Lewis acidic carbodiimide as well as those exhibiting R_3_P=O → Li and R_2_C=O → Li coordinations using the classic Dipp2NacNacLi ligand.

## Introduction

When attached to organic ligands, lithium and magnesium represent the luminaries of the s-block of the periodic table, arguably of the whole of the periodic table, in terms of their phenomenal synthetic utility. It is not surprising therefore that both metals have been involved in the proliferation of the chemistry of β-diketiminate (NacNac or BDI) ligands that has taken place over the past 20 or so years. Though originating in the 1960s,^1−4^ β-diketiminate chemistry took about 30 years before its tunable spectator ligand credentials rose to the fore^5^ in the mold of its cyclopentadienyl (Cp and substituted Cp) predecessors.

Befitting the “masters of mediation” eminence of organic alkali metal compounds in general,^6^ alkali metal β-diketiminates are generally utilized for transferring their dinitrogen ligands to other metals. This was first established with lithium and tin in 1994 by Lappert, with the report also recording one of the first crystal structures of a lithium β-diketiminate.^7^ Today this utilization continues as for example by Kretschmer, who detailed an elegant but simple approach to obtaining aluminum and gallium β-diketiminate complexes from the sodium congener,^8^ as well as by the groups of Schaper and Shen, who used the lithium or sodium β-diketiminate complex in the convenient preparation of zirconium and lanthanide β-diketiminate complexes, respectively.^9−11^

Power’s straightforward high-yielding synthesis of the aminoimine 2,6-diisopropylphenyl-β-methyldiketiminate [NacNac (Me, Dipp) but labeled here for brevity as NacNac(H)]^12^ opened up access to this ligand to a wider community, to the extent that it is still the most popular β-diketiminate proligand utilized today and the subject of this present paper. β-Diketiminate ligands in general have made a particularly strong impact in Group 2.^13−20^ The same β-diketiminate ligand (NacNac) was behind the opening of an exciting new area of Group 2 chemistry through Jones and Stasch’s pioneering of thermally stable magnesium(I) compounds of type (NacNac)MgMg(NacNac).^21^ Other highlights of magnesium β-diketiminate chemistry include advances made in the ring-opening polymerization (ROP) of lactides.^22−25^ First synthesized independently in 2002 by Roesky^26^ and Gibson,^27^ NacNacMg(*n*-Bu) has recently taken on greater significance through Hill’s catalytic and insertion applications.^28^ Surprisingly, given the prominence of *n*-Bu and TMP s-block reagents,^29−32^ the analogous amide, NacNacMg(TMP), was only introduced as recently as 2013 (TMP is 2,2,6,6-tetramethylpiperidide).^33^ This study by Hevia established that NacNacMg(TMP) is a more effective metallating (C–H deprotonating) agent than its alkyl analogue NacNacMg(*n*-Bu), reversing the normal order of alkyl versus amide basicity.

Despite these developments, studies probing the behavior of these s-block NacNac species with small molecules have been relatively scarce, especially for Group 1 examples.^34−38^ Our first expedition in this area found that the electrophilic species CO_2_, *t*-BuNCO, and *i*-PrNCO all reacted with lithium NacNac at the γ-C site of the ligand backbone, instead of at the “frontal” polar Li–N bonds, expelling the innocent, spectator image of alkali-metal-attached NacNac ligands.^39^ Here, the picture becomes more complicated when the outcomes of exposing LiNacNac to carbodiimide and phosphine oxide molecules are revealed. Moreover, bringing NacNacMg(TMP) into this study for the first time by treating it with isocyanate and isothiocyanate molecules reveals surprising reactivities out of kilter with those of conventional Mg TMP-containing compounds.

**Figure 1:**
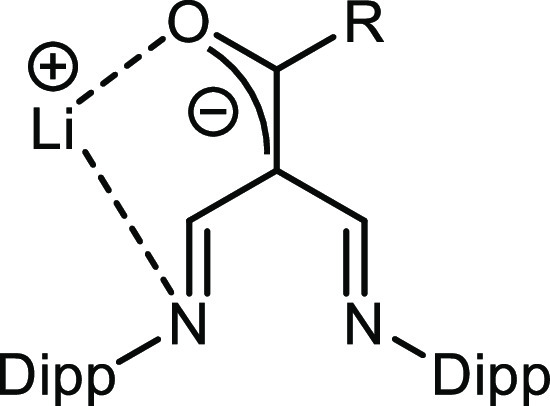
ChemDraw schematic showing general diimine functionality seen when LiNacNac was previously reacted with a series of small molecules, where Dipp is 2,6-diisopropylphenyl.

Since our earlier work on reactions of LiNacNac with unsaturated organic molecules invariably showed backbone γ-carbon reactivity with concomitant redistribution of the NCCCN unit into a diimine, as displayed in Figure 1, we decided to investigate a larger sample of small molecules. Since the NacNac N—C=C bonds can be regarded as joined enamido units, this reactivity at the γ-carbon atoms is not that unexpected.

## Results and Discussion

**Scheme 1:**
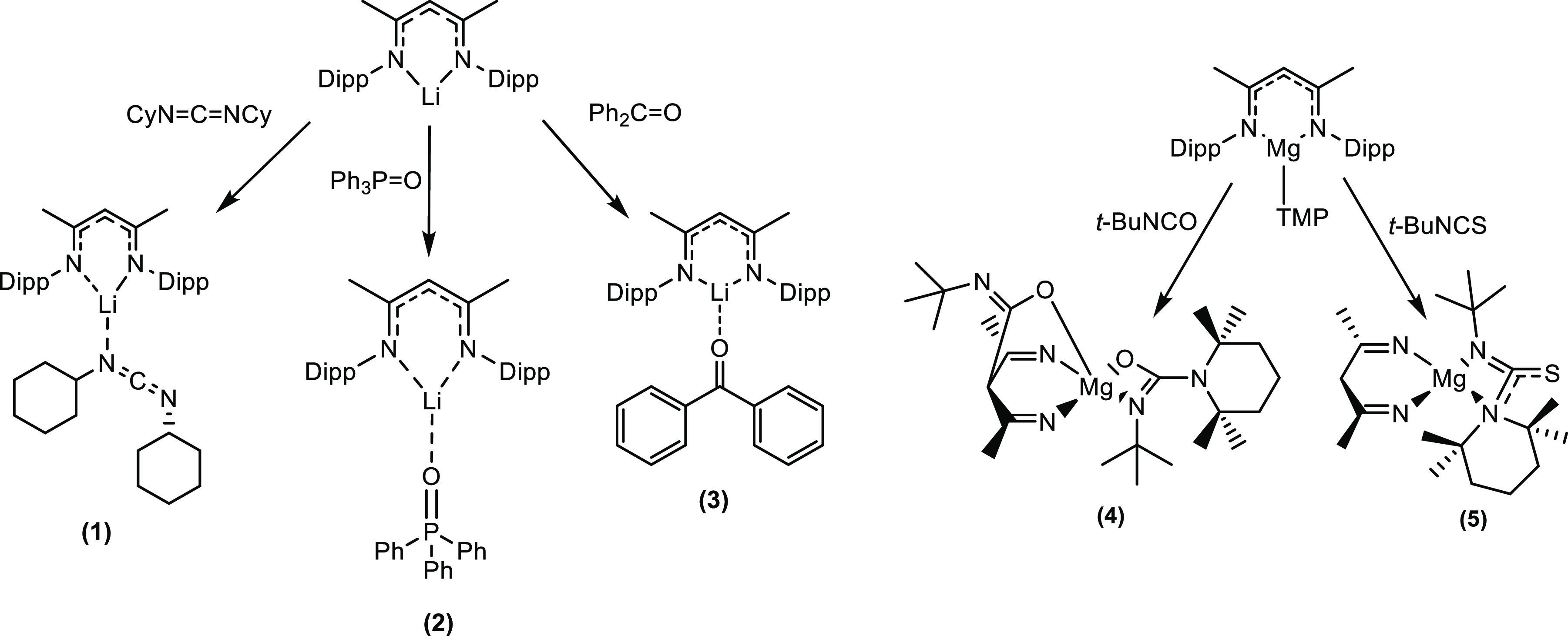
Small Molecule Fixation Reactions Carried out in This Work

Our first choice was *N*,*N*′-dicyclohexylcarbodiimide, CyN=C=NCy, DCC, which has a linear interior akin to that of isoelectronic CO_2_. Thus, a 1:1:1 stoichiometric mixture of LiNacNac, TMEDA (*N*,*N*,*N*′,*N*′-tetramethylethylenediamine), and DCC in hexane solution produced crystals in 83% yield identified by X-ray crystallography as [{(MeCN-2,6-*i*Pr_2_C_6_H_3_)_2_CH}Li·N(Cy)CN(Cy)], **1** (Scheme 1).

**Figure 2:**
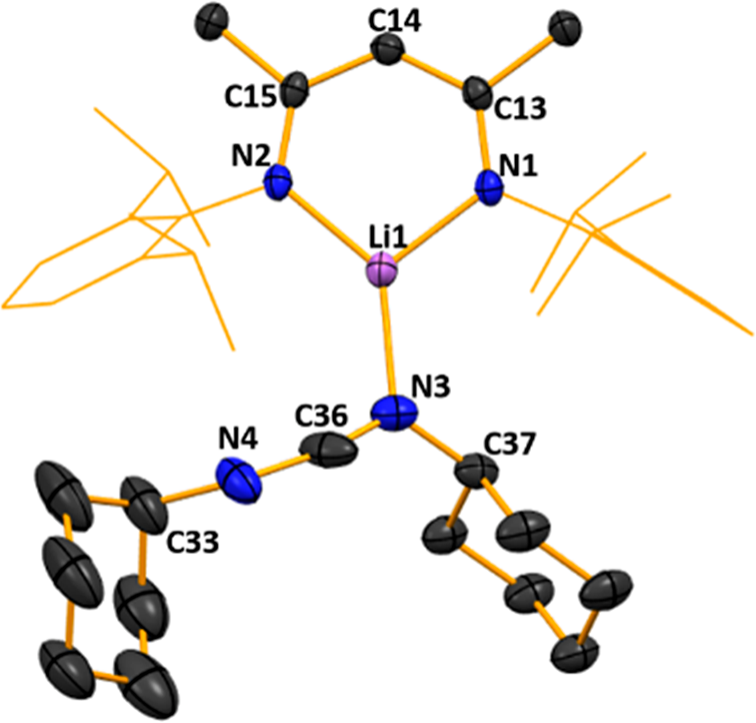
Molecular structure of [{(MeCN-2,6-*i*Pr_2_C_6_H_3_)_2_CH}Li·N(Cy)CN(Cy)] (**1**). H atoms and disorder are omitted and the NacNac Dipp groups are shown as a wire frame for clarity. Thermal ellipsoids are displayed at the 40% probability level.

Its structure (Figure 2) revealed a simple donor–acceptor arrangement with DCC datively attached to the frontal Li atom via one of its terminal atoms (N3). Repeating the reaction but heating the mixture to reflux temperature still afforded **1** as confirmed by NMR spectra of the isolated product, with a ^1^H resonance at 5.02 ppm, corresponding to the still intact backbone γ-hydrogen atom in contrast to the sigmatropic rearrangement of this C—H atom to the N=C bond of the previously studied isocyanate systems.^39^

The planarity of the NCCCN ring in **1** remains intact like those in Power’s Et_2_O and THF LiNacNac structures, while the two C—C and two C—N bond lengths [C—C bond lengths of 1.399(4) Å (C13–C14) and 1.420(4) Å (C14–C15) compared to 1.387(4) Å and 1.418(4) Å respectively in NacNac(H), and C—N bond lengths of 1.324(3) (C13–N1) and 1.318(3) Å (C15–N2) compared to 1.318(4) Å and 1.341(4) Å respectively in NacNac(H)] within the ring become slightly more symmetrical upon lithium substitution compared to NacNac(H), signifying a degree of delocalization of the π-bonding. Lithium exhibits a trigonal planar geometry, with bond angles markedly distorted from idealized values ranging from 100.9(2)° (N2–Li1–N1) to 135.6(3)° (N2–Li1–N3), while also lying effectively equidistant between the two NacNac N atoms [Li1–N1, 1.898(5) Å and Li1–N2, 1.902(5) Å]. Lithium coordination of DCC results in asymmetry in the N=C=N bonds since C36—N3 [1.206(4) Å] is significantly shorter than C36—N4 [1.39(1) Å ]. This distortion is also reflected in the inequivalence of the DCC bond angles [C33—N4=C36, 134.6(8)°] and C37—N3=C36, 121.4(3)°], the former being notably more obtuse than the C—N=C bond angles approaching 120° normally seen in carbodiimide ligands.^40^ The N3–C36–N4 bond angle in **1** also shows a distortion from linearity [165.4(5)°]. These features suggest that the major resonance structure is polarized (Cy)N—C≡N^+^(Cy) rather than (Cy)N=C=N(Cy). Reactions of DCC with organolithium compounds generally follow nucleophilic addition pathways as exemplified by the lithium amidinate FcC(NCy)_2_Li formed when DCC is treated with bulky ferrocenyllithium (FcLi).^41^ There are also several articles referencing such addition reactions between amidinates derived from DCC and organolithium reagents.^42−45^ For example, reacting DCC with LiHMDS leads to amidinate [(Cy)NC{N(SiMe_3_)}N(Cy)·Li], with addition of the N(SiMe_3_) group seen at the central DCC carbon.^46^ However, to the best of our knowledge, there are no crystalline examples of DCC or any other carbodiimide interacting with lithium centers or any other metal centers as a Lewis donor such as that seen in **1**. Thus, **1** can be considered a model intermediate en route to forming an amidinate from a carbodiimide and an alkali metal nucleophilic source. Such κ1-RN=C=NR metal coordinations have been implicated in various catalytic heterofunctionalizations of carbodimides.^47−52^ It has previously been shown by the Harder group that activation of carbodiimides is possible under metal-free conditions, although it was noted that in this case the “extent of activation is less than that in carbodiimide···Li+ complexes”, with harsh conditions necessary under a metal-free environment.^53^ It is likely that the bulk of the DCC molecule prevents formation of an amidinate in this system, with the DCC being too sterically congested to allow for nucleophilic addition of the NacNac in this case. As alluded to earlier, solution NMR data are in agreement with the solid-state structure (see the Supporting Information for full details), suggesting that its composition is maintained in solution. Of note is the ^7^Li spectrum, which showed a single resonance corresponding to the single lithium environment present within **1** at 2.61 ppm. This contrasts with the ^7^Li spectrum of LiNacNac, which shows a resonance at 0.73 ppm, confirming that the lithium in **1** remains in a different environment, due to the donating role of the DCC molecule being retained in solution.

**Figure 3:**
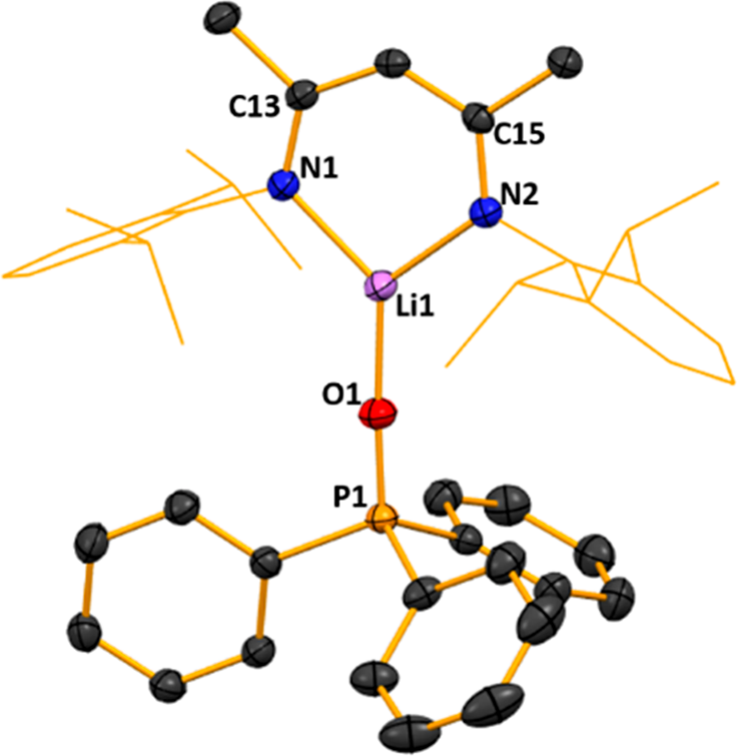
Molecular structure of [{(MeCN-2,6-*i*Pr_2_C_6_H_3_)_2_CH}Li·OP(Ph)_3_] (**2**). H atoms, disorder, and cocrystallized hexane solvent are omitted and the NacNac Dipp groups are shown as a wire frame for clarity. Thermal ellipsoids are displayed at the 40% probability level.

Next, we studied triphenylphosphine oxide, Ph_3_P=O. A 1:1:1 stoichiometric mixture of LiNacNac, Ph_3_P=O and PMDETA (*N*,*N*,*N*′,*N*″,*N*″-pentamethyldiethylenetriamine; added to enhance solubility) in hexane solution deposited crystals (78% yield) identified by X-ray crystallography as [{(MeCN-2,6-*i*Pr_2_C_6_H_3_)_2_CH}Li·OP(Ph)_3_], **2**. Matching that of **1**, the structure of **2** (Figure 3) is a donor–acceptor complex connected via a frontal Li–O bond [1.489(1) Å], with no backbone insertion present.

PMDETA is also absent from **2**, though subsequent experiments showed that PMDETA addition is necessary in order to obtain crystals, although why PMDETA should aid the crystallization process is not yet clear. Without adding PMDETA, **2** can still be made as an amorphous powder, with its identity confirmed by NMR characterization. Occupying a highly distorted trigonal planar NNO coordination, with bond angles ranging from 99.8(2)° (N1–Li1–N2) to 131.4(2)° (O1–Li1–N1), Li lies equidistant between the NacNac N atoms [N1–Li1, 1.925(3) Å and N2–Li1, 1.911(3) Å]. The P–O–Li unit sits exactly within the NCCCN plane, which in turn is not disturbed from planarity, with bond lengths [N1–C13, 1.318(2) Å; C13–C14, 1.407(2) Å; C14–C15, 1.412(2) Å; N2–C15, 1.316(2) Å] indicating a degree of π-delocalization. Multinuclear NMR spectroscopic data on **2** concur with the solid-state structure, particularly showing that coordination between the phosphine oxide and LiNacNac is maintained in solution. This is deduced from the ^7^Li NMR spectrum, which shows a resonance at 2.70 ppm, in contrast to that seen for LiNacNac at 0.73 ppm, indicating a change in the lithium environment. The ^31^P{^1^H} NMR also indicates coordination, with the resonance at 23.2 ppm for uncoordinated triphenylphosphine oxide contrasting to that at 30.80 ppm seen in **2**. Though a CSD search revealed 20 hits for Ph_3_P=O → Li dative bonds, no hits were found for any LiNacNac scaffold, with 7 of the 20 hits featuring a cationic (Ph_3_PO)_4_Li^+^ unit with a balancing counteranion present. Of note is work by Lichtenberg, who structurally characterized a lithium aminotroponiminate (LiATI) solvated by Ph_3_PO, exhibiting a Li chelated by two ATI N atoms in a similar arrangement to that of **2** but with an additional O (THF) ligation.^54^ Significantly, a search of the CSD revealed no hits for a phosphine oxide unit bonded to LiNacNac, with the closest match being a 1,8-C_10_H_6_{NHSiMe_3_}_2_-supported dilithium compound; however, the lack of delocalization over the backbone of this ligand limits comparison.^55^ Attempted reactions with the sulfur analogue Ph_3_P=S failed to produce a complex with LiNacNac as determined via NMR studies, contrasting with Nikonov’s report of NacNacAl(I) which forms NacNacAl(=S)S=PPh_3_ via a complexation/oxidative cleavage process.^56^

**Figure 4:**
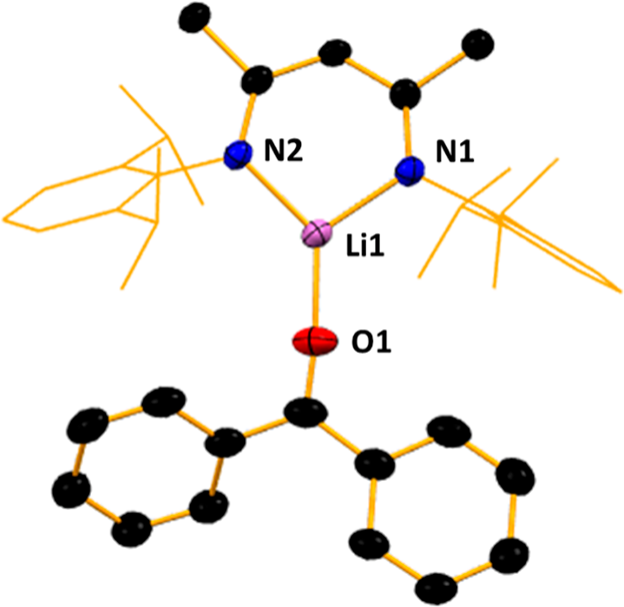
Molecular structure of [{(MeCN-2,6-*i*Pr_2_C_6_H_3_)_2_CH}Li·OC(Ph)_2_] (**3**). H atoms are omitted and the NacNac Dipp groups are shown as a wire frame for clarity. Thermal ellipsoids are displayed at the 40% probability level.

Our third and final homoleptic LiNacNac structure was obtained from the reaction between the ketone benzophenone and LiNacNac. A mixture of LiNacNac and a slight stoichiometric excess of benzophenone (1:1.25) was reacted in hexane, with two drops of PMDETA (*N*,*N*,*N*′,*N*″,*N*″-pentamethyldiethylenetriamine) added for crystallization purposes. This solution deposited a crop of large red crystals (in a 59% yield) identified by X-ray crystallography as [{(MeCN-2,6-*i*Pr_2_C_6_H_3_)_2_CH}Li·OC(Ph)_2_], **3**. Following the trend set by **1** and **2**, the structure of **3** (Figure 4) is yet another donor–acceptor complex, connected via a slightly elongated frontal Li–O bond [1.843(3) Å], when compared to that observed in compound **2**. Of note is that once again no backbone insertion at the γ-carbon is observed, in contrast to that noted in previous work.^39^

In a similar manner to **2**, PMDETA addition was shown to be required for crystallization of compound **3**, despite it not being part of the crystal structure obtained. The lithium atom of compound **3** sits in an approximately trigonal planar coordination environment, with bond angles ranging from 101.22(14)° (N1–Li1–N2) to 133.92(18)° (O1–Li1–N1). The lithium center lies equidistant between the NacNac N atoms [N1–Li1, 1.908(3) Å and N2–Li1, 1.914(3) Å]. Akin to that in compound **2**, the C–O–Li unit of compound **3** sits within the NCCCN plane, which retains its planarity. Bond lengths [N1–C5, 1.326(2) Å; C5–C6, 1.410(2) Å; C6–C4, 1.408(2) Å; N2–C4, 1.317(2) Å] are also indicative of a degree of π-delocalization. Multinuclear NMR spectroscopic data on **3** concurred with the solid-state structure, particularly showing that coordination between the benzophenone and LiNacNac is maintained in solution. This is deduced from the ^7^Li NMR spectrum, which shows a resonance at 3.64 ppm, in contrast to that seen for LiNacNac at 0.79 ppm, indicating a significant change in the lithium environment.

**Scheme 2:**
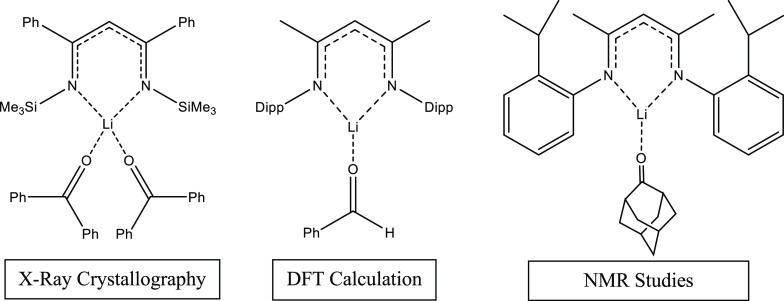
Selection of Products Found Involving C=O Bonds Interacting with Various β-Diketiminate Scaffolds

Upon searching the CSD, it was discovered there were no examples of bonding between Ph_2_C=O and Li involving the extensively studied Dipp_2_NacNac scaffold. In fact, there were no examples at all of an aldehyde/ketone C=O unit coordinating to either Li, Na, or K within the Dipp_2_NacNac scaffold in this way, with examples of benzophenone coordinating to lithium metal in any environment being rare.^57,58^ However, there was a single hit for dative Ph_2_C=O → LiNacNac bonding featuring a much less commonly studied variant of NacNac, [Me_3_SiNC(Ph)CHC(Ph)NSiMe_3_]Li] by Tong and Liu.^59^ This unusual ligand (displayed in Scheme 2) could be viewed as an inversion of the ubiquitous Dipp_2_NacNac ligand, as now we see a rich electron donor sitting on the α-nitrogen position while an electron withdrawing group sits on the β-carbon positions. The presence of phenyl rings on the backbone, acting as electron withdrawing groups, may also alter the nucleophilicity of the γ-carbon position, a key feature in several recent small molecule activations using the Dipp_2_NacNac scaffold.^37^

The contrast with this 2008 structure can help rationalize the dominance of the Dipp_2_NacNac ligand, with the Dipp groups flanking the metal allowing for coverage of the coordination sphere to be maximized which helps to prevent coordination to multiple reactant molecules, unwanted solvent interactions, or dimerization. The dual coordination of benzophenone units in [[Me_3_SiNC(Ph)CHC(Ph)NSiMe_3_]Li] can thus be rationalized both by the reduction of steric bulk around the metal center decreasing the activation barrier and the loss of steric coverage of the coordination sphere.

Interestingly, Sen et al. recently reported that LiNacNac could catalyze hydroboration reactions of aldehydes and ketones using pinacolborane, theorizing a catalytic cycle involving a donor–acceptor Ph(H)C=O → LiNacNac intermediate.^60^ While complex **3** incorporates benzophenone rather than benzaldehyde, it exemplifies that the RC=O → LiNacNac unit predicted by Sen et al. using DFT calculations is achievable. In view of the aforementioned inverted NacNac structure reported by Liu, this may also highlight the advantage of Dipp_2_NacNac in preventing saturation of the metal coordination sphere, facilitating the formation of catalytically active intermediates such as this. Meanwhile complex **2** could be viewed as a model intermediate of this catalysis with P=O as opposed to C=O coordination. The Mair group similarly predicted an intermediate containing the RC=O → LiNacNac unit in their 2003 paper on reversible C—C bond formation.^61^ On this occasion NMR evidence was cited to suggest the presence of the adamantanone-coordinated intermediate structure, supported by a 1,5-diazapentadienyllithium complex, a close cousin of Dipp_2_NacNac, with only one less iso-propyl arm per phenyl ring. This new structure is evidence that such structures are obtainable in the solid state, as well as being observed by NMR in the solution state.

These three results with DCC, Ph_3_P=O, and Ph_2_C=O show that coordination of bulky nitrogen and oxygen donors to LiNacNac is possible, although for these donor systems coordination occurs preferentially at the frontal site of the molecule, with the NacNac backbone remaining undisturbed; this is in contrast with results obtained with isocyanates and CO_2_, where nucleophilic attack occurs via the backbone γ-carbon position.^39^

**Scheme 3:**
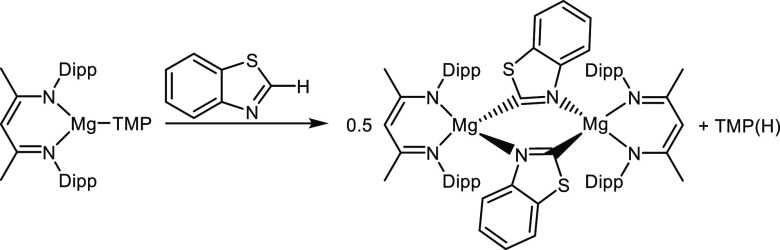
Trapping of Benzothiazole Using NacNacMg(TMP), with Concomitant Formation of TMP(H), as Reported by Hevia^33^

As alluded to earlier, Hevia’s work has established that heteroleptic NacNacMg(TMP) is an efficient TMP base for selectively deprotonating sensitive organic molecules such as 1,3-benzoazoles or fluorinated aromatic compounds, capturing the emergent anionic molecules and concomitantly releasing TMP(H), as shown in Scheme 3.^33^

**Figure 5:**
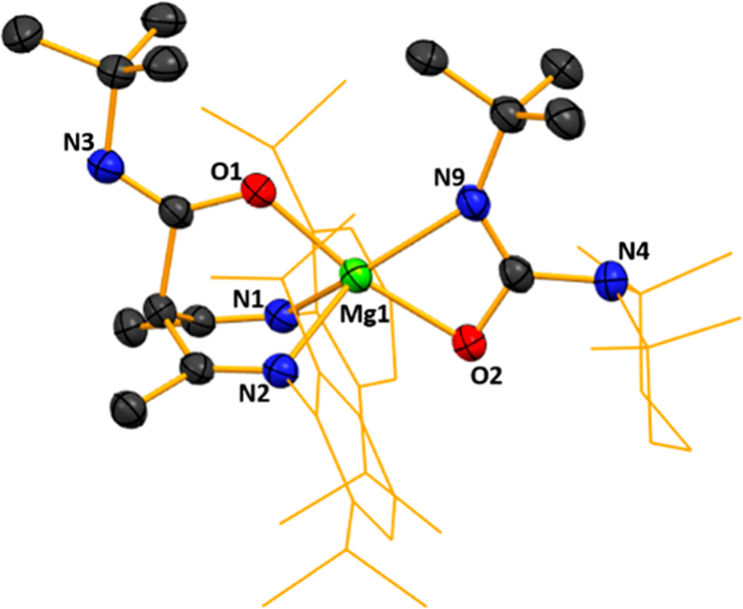
Molecular structure of [{(MeCN-2,6-*i*Pr_2_C_6_H_3_)_2_CH(CON(*t*-Bu)}Mg(CON(*t*-Bu))TMP] (**4**). H atoms and cocrystallized hexane solvent are omitted and NacNac Dipp groups and organic TMP scaffold are shown as a wire frame for clarity. Thermal ellipsoids are displayed at the 40% probability level.

Mg(TMP) systems are good bases in general though they tend to work best in synergistically operative bimetallic mixtures.^62−65^ These reactions piqued our curiosity so we decided to also investigate the small molecule chemistry of NacNacMg(TMP) with molecules bereft of acidic bonds, namely with isocyanate *t*-BuNCO and isothiocyanate *t*-BuNCS (Scheme 1). Surprisingly, with *t*-BuNCO, a 2-fold insertion was seen, with one isocyanate molecule inserting into the γ-C site, while another inserted into the Mg–N(TMP) bond. No insertion was seen at a frontal Mg–N(NacNac) site. Evident from the crystal structure of the isolated urea-type product, [{(MeCN-2,6-*i*Pr_2_C_6_H_3_)_2_CH(CON(*t*-Bu)}Mg(CON(*t*-Bu))TMP] **4** (Figure 5), this 2-fold reactivity contrasts with that of LiNacNac with *t*-BuNCO, where a single insertion occurred at the γ-C site. When this NacNacMg(TMP) reaction was repeated rationally with two equivalents of *t*-BuNCO instead of one, the same product was formed, proving the reproducibility of the reaction, but only in a similarly small yield. These low isolated yields can be attributed to the high solubility of **4** in nonpolar aprotic solvents. The standout feature of **4** is TMP acting as a nucleophile, as its inherent low nucleophilicity is its prized asset with regard to its popularity as a selective base with unsaturated substrates, though rare examples of TMP nucleophilicity exist.^66^ Another feature of interest is that there is no sigmatropic hydride rearrangement of a hydrogen from the γ-carbon to the nitrogen of either isocyanate unit, as seen in the lithium NacNac case, though there is concomitant redistribution of the NacNac NCCCN unit into a diimine as seen previously. In **4**, Mg occupies a distorted trigonal bipyramidal site comprising two Mg–(axial)O bonds (O1–Mg1–O2 bond angle, 169.7(1)°) and three Mg–(equatorial)N bonds (average N–Mg–N bond angle, 119°). The C–N and C–O bond lengths of approximately 1.3 (Å) support the hypothesis of two highly delocalized systems within the NCO unit, with both bonds existing as intermediate between single and double bonds. Solution-state NMR characterization of **4** was also carried out (see the SI for full details).

**Figure 6:**
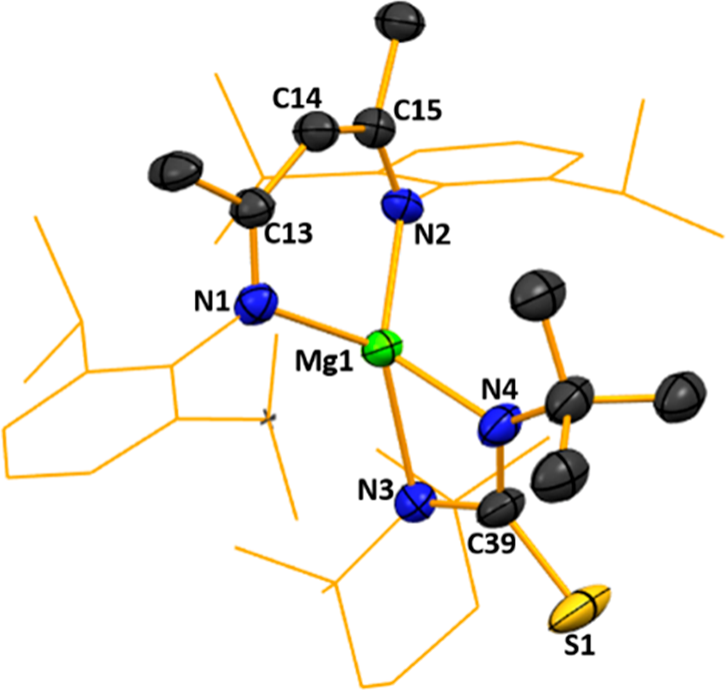
Molecular structure of [{(MeCN-2,6-*i*Pr)_2_C_6_H_3_)_2_CH}Mg(TMP)(*t*-BuNCS)] (**5**). H atoms, disorder, and a second molecule are omitted and the NacNac Dipp groups and organic TMP scaffold are shown as a wire frame for clarity. Thermal ellipsoids are displayed at the 40% probability level.

The final small molecule studied was isothiocyanate *t*-BuNCS, prompted by a recent report by Ma et al., who found that the NacNacMg(*n*-Bu) dimer exhibited different reactivities depending on the ratio of PhNCS used.^34^ Exposure to one equivalent led to deaggregation of the dimer and bidentate (N, S) coordination to Mg via a thioamidinate NC(*n*-Bu)S unit, while exposure to two equivalents showed this coordination again but combined with coordination at the NacNac backbone, similar to that found in **4**. Since our attempts to grow a crystalline product from NacNacMg(TMP) and PhNCS failed, we turned to *t*-BuNCS. Surprisingly, neither of these aforementioned coordinations were found in [{(MeCN-2,6-*i*Pr)_2_C_6_H_3_)_2_CH}Mg(TMP)(*t*-BuNCS)] **5**, the product of the 1:1 NacNacMg(TMP) and *t*-BuNCS reaction. Instead, the structure of **5** (Figure 6) shows addition of the Mg–N(TMP) bond to the NC bond of the NCS unit to form a four-membered MgNCN ring, leaving the sulfur uncoordinated to the Mg.

Thus, the TMP unit again plays an unfamiliar addition role as in **4** but in a different way as it is now bridging as opposed to terminal, a distinction dictated by the different heteroatoms on the cyanate units with Mg preferring to bind to O over N in the case of **4** and to N over S in the case of **5**, keeping with the HSAB concept.^67^ This basic concept can also rationalize the different N, S coordination observed in the Ma structure, as Mg has less affinity for the adding *n*-Bu group than the S heteroatom. There are two crystallographically independent molecules within the unit cell of **5**; however, as the metrics for each molecule are essentially identical, only one is discussed. Centralized magnesium occupies a highly distorted tetrahedral environment, with bond angles ranging from 63.4(2)° for N3–Mg1–N4 to 128.0(2)° for N1–Mg1–N3. In **3**, the N1–Mg1–N2 chelation angle is 88.2(1)°, while in **5** it increases to 94.8(2)°. It also lies equidistant between the chelating nitrogen atoms of the NacNac ligand in a similar situation to that seen in **4** [N1–Mg1, 2.097(5) Å; N2–Mg1, 2.105(5) Å; cf. N1–Mg1 2.162(3) Å; N2–Mg1 2.163(2) Å in **3**]. The NacNac ligand is essentially planar, with the root-mean-square (RMS) deviation from linearity being less than 1 Å [0.075(4) Å], with Mg lying 0.744(6) Å outside this plane. Bonding in the NC(NTMP)S unit appears to be that of an TMP-based imine unit, based on the bond lengths of N3–C39 [1.506(9) Å) and N4–C39 (1.297(9) Å], with a C–S bond length of 1.701(7) Å. These lengths suggest considerable delocalization within this NC(NTMP)S unit (specifically across S1–C39–N3), which adopts a thiourea-like arrangement.

## Conclusions

We have demonstrated that there remains a significant and wide variety of potential reactivities of homoleptic LiNacNac and heteroleptic NacNacMg(TMP) β-diketiminates toward small unsaturated molecules that are yet to be fully explored. In this study we have structurally characterized what is, to the best of our knowledge, the first example of a carbodiimide acting as a Lewis donor toward an alkali metal center, as well as the first structural example of a phosphine oxide binding to LiNacNac. In contrast to our previous work, both products display reactivity at the front of the NacNac scaffold, a feature that was also found with benzophenone. The benzophenone structure could act as a model compound for the binding of an aldehyde or ketone to the LiNacNac scaffold. Further exemplifying the unusual reactivity that we have come across in NacNac chemistry, we have shown the unconventional behavior of the typically selective base TMP, in which we see a rare case of TMP acting as a nucleophile in order to play an addition role, e.g., in one example in a terminal fashion and bridging in another, in a manner which is rarely seen, though in keeping with classical HSAB theory. One facet of future work is to determine what effect changing the alkali metal of the whole of Group 1 (Li–Cs) will have on the structure and reactivity of NacNac and related compounds.^6^ Future work will also consider the development of new NacNac-derived ligands with extra functionality such as that displayed in the urea-like compound **4**.

## Supporting Information Available

Experimental methods, crystallographic information, NMR spectra, IR data, and melting point data (PDF)

## Accession Codes

CCDC 2061662–2061666 contain the supplementary crystallographic data for this paper. These data can be obtained free of charge via www.ccdc.cam.ac.uk/data_request/cif, or by emailing data_request@ccdc.cam.ac.uk, or by contacting The Cambridge Crystallographic Data Centre, 12 Union Road, Cambridge CB2 1EZ, UK; fax: +44 1223 336033.
