# Supplementary material for: Expected and Unexpected Reactivities of Homoleptic LiNacNac and Heteroleptic NacNacMg(TMP) β-Diketiminates toward Various Small Unsaturated Organic Molecules
Source: Inorg Chem. 2021 Apr 8;60(8):6057–64. doi: 10.1021/acs.inorgchem.1c00549 (PMC8154426; doi:10.1021/acs.inorgchem.1c00549)
Supplement: Supplementary file 1 — ic1c00549_si_001.pdf [file ic1c00549_si_001.pdf]

# Supporting Information to accompany

## Expected and unexpected reactivities of homoleptic LiNacNac and heteroleptic NacNacMg(TMP) β-diketiminates towards various small unsaturated organic molecules

*Richard M. Gauld,‡†[a] Jennifer R. Lynch,‡[a] Alan R. Kennedy,[a] Jim Barker,[b] Jacqueline Reid,[b] and Robert E. Mulvey*[a]*

a. WestCHEM, Department of Pure and Applied Chemistry, University of Strathclyde, Glasgow, G1 1XL, UK.

b. Innospec Ltd., Innospec Manufacturing Park, Oil Sites Road, Ellesmere Port, Cheshire, CH65 4EY, UK.

† Faculty of Chemistry and Biochemistry, Ruhr University Bochum, Universitätsstrasse 150, 44780, Bochum, Germany.

e-mail: r.e.mulvey@strath.ac.uk

# Contents

General Experimental ........ 3

X-ray crystallography ........ 3

Synthesis of [{(MeCN-2,6-$^{i}Pr_2C_6H_3)_2$CH}Li.N(Cy)CN(Cy)] (**1**) ........ 5

Figure S1: ORTEP diagram of [{(MeCN-2,6-$^{i}Pr_2C_6H_3)_2$CH}Li.N(Cy)CN(Cy)] (**1**) ........ 6

Synthesis of [{(MeCN-2,6-$^{i}Pr_2C_6H_3)_2$CH}Li.OP(Ph)$_3$] (**2**) ........ 9

Figure S2: ORTEP diagram of [{(MeCN-2,6-$^{i}Pr_2C_6H_3)_2$CH}Li.OP(Ph)$_3$] (**2**) ........ 10

Synthesis of [{(MeCN-2,6-$^{i}Pr_2C_6H_3)_2$CH}Li.OC(Ph)$_2$] (**3**) ........ 13

Figure S3: ORTEP diagram of [{(MeCN-2,6-$^{i}Pr_2C_6H_3)_2$CH}Li.OC(Ph)$_2$] (**3**) ........ 14

Synthesis of [{(MeCN-2,6-$^{i}Pr_2C_6H_3)_2$CH(CON($^{t}$Bu)}Mg(CON($^{t}$Bu))TMP] (**4**) ........ 17

Figure S4: ORTEP diagram of [{(MeCN-2,6-$^{i}Pr_2C_6H_3)_2$CH(CON($^{t}$Bu)}Mg(CON($^{t}$Bu))TMP] (**4**) ........ 18

Synthesis of [{(MeCN-2,6-$^{i}$Pr)$_2C_6H_3)_2$CH}Mg(TMP)($^{t}$BuNCS)] (**5**) ........ 20

Figure S5: ORTEP diagram of [{(MeCN-2,6-$^{i}$Pr)$_2C_6H_3)_2$CH}Mg(TMP)($^{t}$BuNCS)] (**5**) ........ 21

Table 1: X-ray crystal structural data and refinement details for compounds 1-5 ........ 23

References ........ 25

# General Experimental

All reactions were performed under a protective argon or nitrogen atmosphere using either standard Schlenk or glove box techniques. Hexane and THF were dried by heating to reflux over sodium benzophenone ketyl and then distilled under nitrogen prior to use. $C_6D_6$ and $d_8$-THF were degassed by freeze-pump-thaw methods and stored over activated 4 Å molecular sieves. $^n$BuLi (1.6 M in hexane) was purchased commercially from Sigma-Aldrich and used as received. TMEDA (N,N,N',N'-tetramethylethylenediamine) was purchased from Sigma-Aldrich, distilled under nitrogen and stored over activated 4 Å molecular sieves prior to use. The parent NacNac(H) ligand and magnesium derivative NacNacMgTMP were made *via* literature procedures.[1,2] Isocyanates *t*-BuNCO and *i*-PrNCO were purchased from Sigma-Aldrich, stored in the refrigerator and used as received. N, N'-dicyclohexylcarbodiimide (DCC) and triphenylphosphine oxide ($Ph_3PO$) were purchased from Fluorochem, while benzophenone (Ph2CO) was purchased from Alfa Aesar, all were used as received. All other reagents were purchased from commercial sources and were also used as received.

NMR spectra were recorded on a Bruker AV3 or AV400 MHz spectrometer operating 400.13 MHz for $^1$H, 155.47 MHz for $^7$Li, 162.0 MHz for $^{31}$P and 100.62 MHz for $^{13}$C. All $^{13}$C NMR spectra were proton decoupled. $^1$H, $^{13}$C{$^1$H}, $^7$Li and $^{31}$P chemical shifts are expressed in parts per million (δ, ppm) and where appropriate referenced to residual solvent peaks or external references.

Melting points were determined in sealed argon filled capillaries and were not corrected.

All Infra-Red analysis was carried out on a PerkinElmer Spectrum 100 Optica FT-IR Spectrometer with solid state samples being prepared in the glovebox using Nujol Mull.

# X-ray crystallography

Data for complexes **1**-**5** were collected on an Oxford Diffraction Gemini S instrument with graphite-monochromated Cu Kα (λ 1.54184 Å) radiation. Data collection and processing used CrysalisPro software.[3] All structures were solved and refined to convergence on $F^2$ against all independent reflections by the full-matrix least squares method using SHELXL[4] as implemented within OLEX2.[5] For **1** disordered and partially present solvent, believed to be hexane, could not be modelled properly. This was dealt with using the SQUEEZE routine as implemented in PLATON.[6] A total of 62 electron equivalents were removed from 312 Å$^3$ of each unit cell's volume. Samples of **4** were found to be twinned. The model adopted was refined against an hklf 5 formatted dataset produced by CrysalisPro. The scale factor refined to 0.7453(10):0.2547(10). Disordered groups were each modelled over two sites with appropriate restraints and constraints applied to bond lengths and displacement ellipsoids

to ensure approximation to expected behaviour. The groups modelled in this way were, one NCy group of **1**, and single $^{i}$Pr groups inboth **2** and **5**. Selected bond lengths and angles are presented in Figures S1-S5, selected crystallographic data are shown in Table 1. Deposition Numbers 2061662-2061666 contain the full supplementary crystallographic data for this paper in cif format. These data are provided free of charge by the joint Cambridge Crystallographic Data Centre and Fachinformationszentrum Karlsruhe Access Structures service www.ccdc.cam.ac.uk/structures.

## Synthesis of [{(MeCN-2,6-$^{i}Pr_2C_6H_3)_2$CH}Li.N(Cy)CN(Cy)] (**1**)

NacNac(H) (1 mmol, 0.41 g) was added to a Schlenk flask and dissolved in hexane (5 ml). This solution was cooled to 0 °C following which $^{n}$BuLi (1.1 mmol, 0.72 ml) and TMEDA (1 mmol, 0.15 ml) were added to give a yellow solution. Next, DCC (1 mmol, 0.206 g) was introduced and the yellow solution was concentrated and cooled in the refrigerator. This cold solution deposited small colorless crystals within 24 hours (yield 0.523 g, 0.83 mmol, 83 %).

**$^{1}$H NMR (400.1 MHz, $C_6D_6$, 300 K): δ** 7.12 (d, 4H, C*H*, Ar*), 7.02 (t, 2H, C*H*, Ar*), 5.02 (s, 1H, C*H*, γ-CH), 3.43 (pent, 4H, C*H*, $^{i}$Pr), 2.27 (s, 2H, C*H*, DCC), 1.88 (s, 6H, C$H_3$, Me), 1.56 (s, 5H, C$H_2$, DCC), 1.44 (s, 5H, C$H_2$, DCC), 1.27 (quart, 26H, C$H_3$, $^{i}$Pr) ppm.

**$^{13}$C{$^{1}$H} NMR (100.6 MHz, $C_6D_6$, 300 K): δ** 163.8 (*C*-CH$_3$, NacNac), 141.1 (*C*H, Ar*), 123.6 ($C_{quaternary,}$ Ar*), 123.3 ($C_{quaternary}$, Ar*), 122.9 ($C_{quaternary}$, Ar*), 93.2 (*C*H, NacNac), 55.8 (*C*H, TMEDA), 35.0 (*C*H, TMEDA), 28.3 (*C*H, $^{i}$Pr), 24.6 (*C*H, $^{i}$Pr), 23.7 (*C*H, $^{i}$Pr), 14.3 (*C*H$_3$, NacNac) ppm.

**$^{7}$Li NMR (155.5 MHz, $C_6D_6$, 300K): δ** 2.61 (s) ppm.

**IR spectrum:** Analysis run in Nujol mull using solid state FT-IR **γ** ~2122 (carbodiimide N=C=N stretching), ~1730 (imine C=N stretching), ~1643 (imine C=N stretching) cm$^{-1}$.

**Elemental analysis:** Due to the air- and moisture-sensitivity of this compound accurate elemental analysis could not be obtained.

**Melting point analysis**: Compound decomposed to a gel over the range of 44 °C – 65 °C and became a liquid at ~72 °C.

## Figure S1: ORTEP diagram of [{(MeCN-2,6-$^{i}$Pr$_2$C$_6$H$_3$)$_2$CH}Li.N(Cy)CN(Cy)] (1)

There are two crystallographically independent molecules featured in the structure, though only one is presented. Hydrogen atoms omitted for clarity. Organic part of DippNacNac ligand shown as wireframe for clarity.

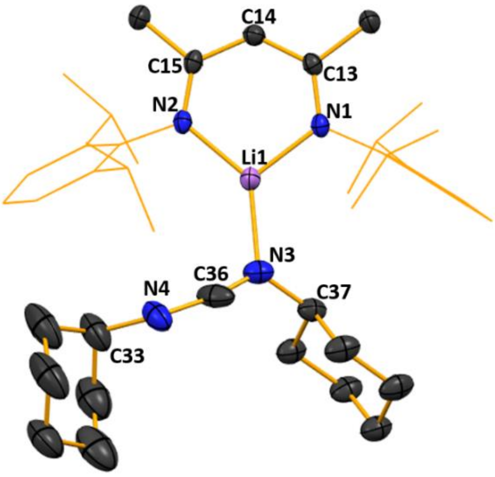


**Selected bond lengths (Å) and bond angles (°)**

Li1 N1 1.898(4), Li1 N2 1.902(5), Li1 N3 2.031(5), N2 C15 1.318(3), C15 C14 1.420(3) C14 C13 1.399(3) C13 N1 1.324(3) C37 N3 1.485(4) C33 N4 1.469(5), N4 C36 1.394(9) C36 N3 1.205(4).

N2 Li1 N1 101.4(2), N2 Li1 N3 135.5(2) N1 Li1 N3 123.0(2) Li1 N3 C36 116.4(3) Li1 N3 C37 122.1(2) N4 C36 N3 172.0(3).

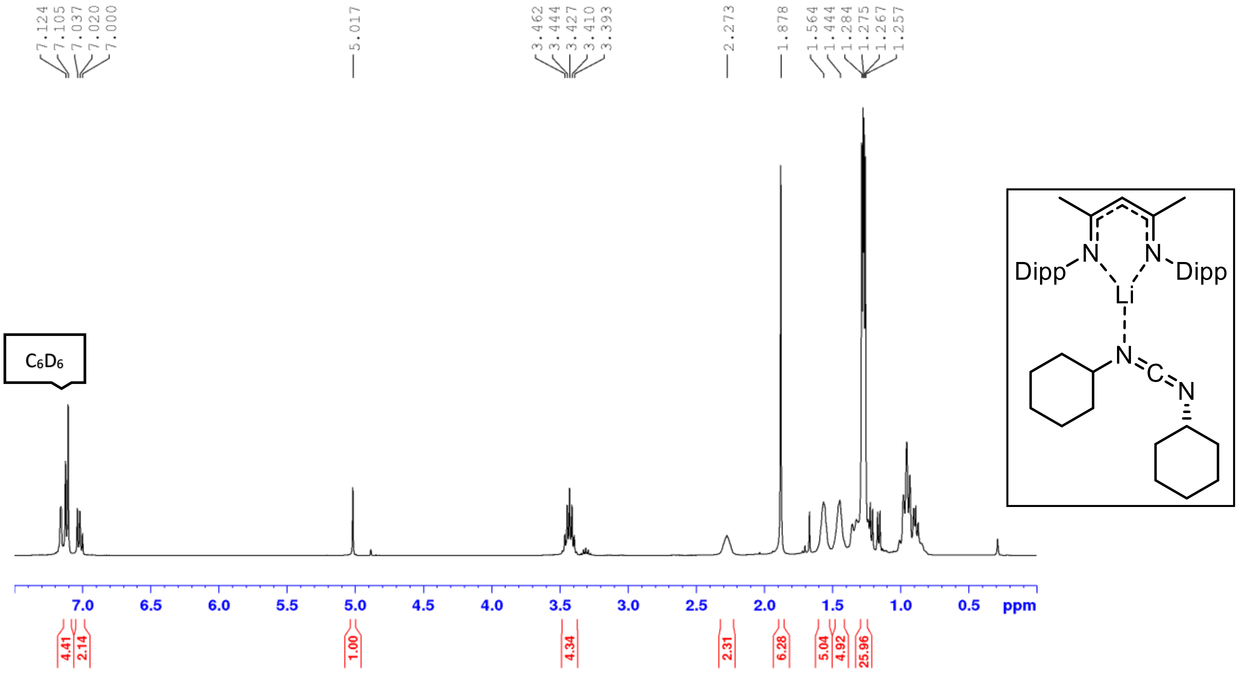


**Figure S1.1**: $^{1}$H NMR spectrum in C$_6$D$_6$ of **1** [{(MeCN-2,6-iPr$_2$C$_6$H$_3$)$_2$CH}Li.N(Cy)CN(Cy)]

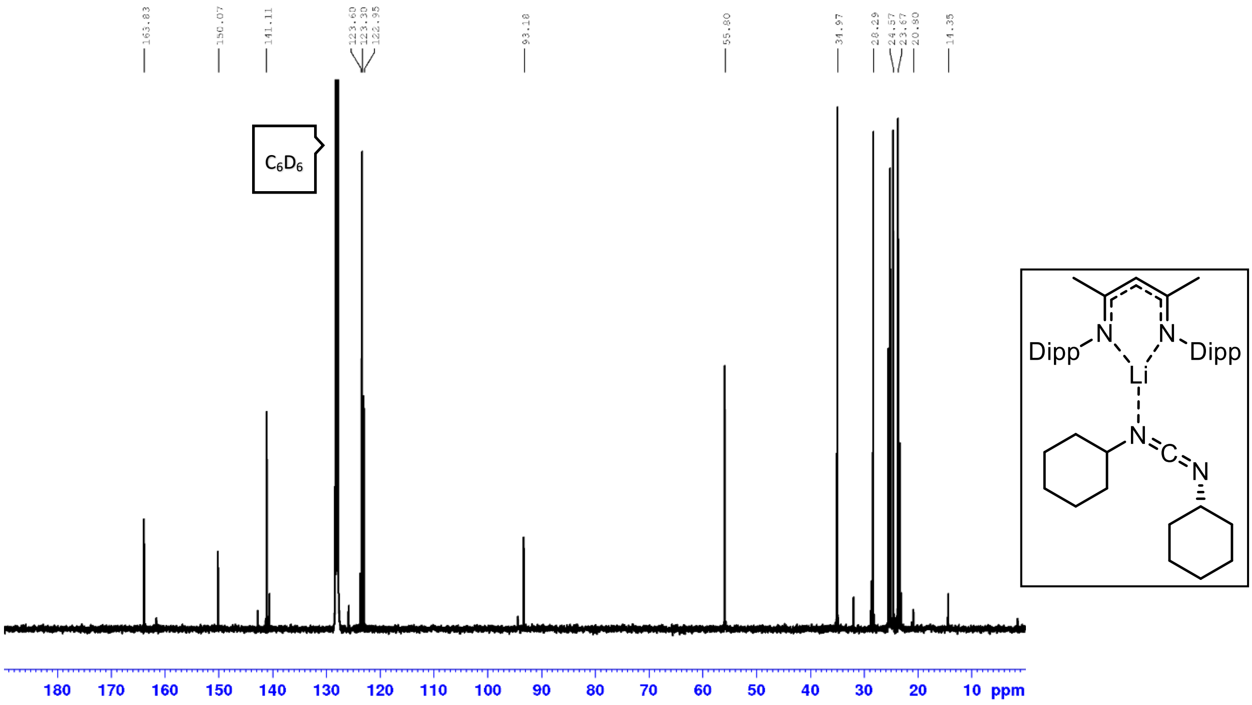


**Figure S1.2**: $^{13}C\{^{1}H\}$ NMR spectrum in $C_6D_6$ of **1** [{(MeCN-2,6-iPr$_2$C$_6$H$_3$)$_2$CH}Li.N(Cy)CN(Cy)]

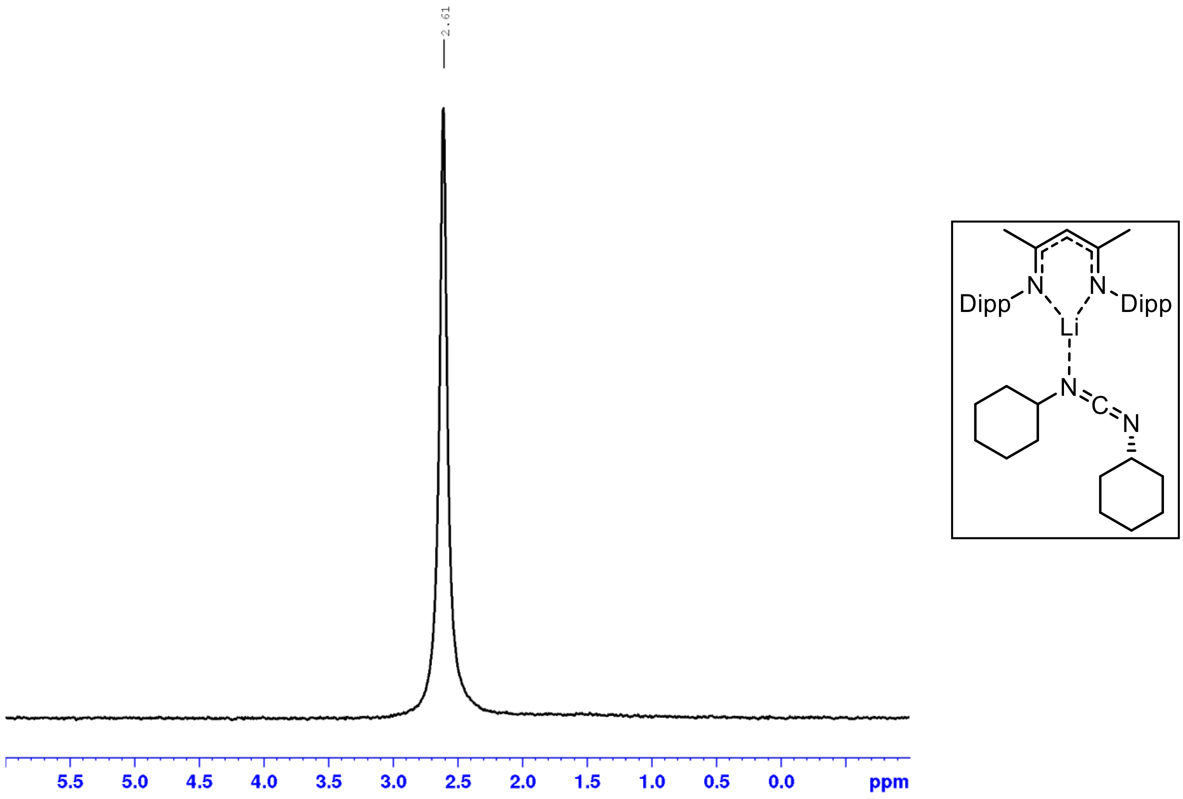


**Figure S1.3**: $^{7}Li$ NMR spectrum in $C_6D_6$ of **1** [{(MeCN-2,6-iPr$_2$C$_6$H$_3$)$_2$CH}Li.N(Cy)CN(Cy)]

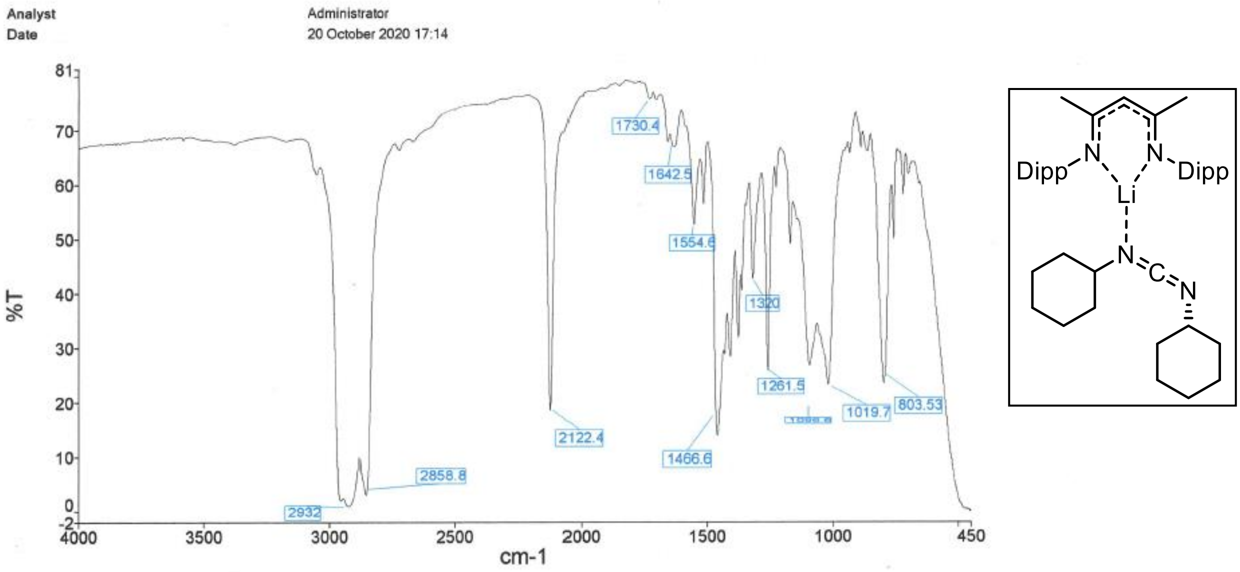


**Figure S1.4**: FT-IR spectrum of compound **1** [{(MeCN-2,6-iPr$_2$C$_6$H$_3$)$_2$CH}Li.N(Cy)CN(Cy)]

## Synthesis of [{(MeCN-2,6-$^i$Pr$_2$C$_6$H$_3$)$_2$CH}Li.OP(Ph)$_3$] (2)

NacNac(H) (1 mmol, 0.41 g) was added to a Schlenk flask and dissolved in hexane (5 ml). This solution was cooled to 0 °C following which $^n$BuLi (1.1 mmol, 0.75 ml) and PMDETA (1 mmol, 0.21 ml) were added to give a yellow solution. Ph$_3$PO (1 mmol, 0.278 g) was then introduced, turning the solution orange. Next the solution was concentrated in vacuo and cooled to -18 °C. This produced formed small colorless crystals within 24 hours (yield 0.55 g, 0.78 mmol, 78 %).

**$^1$H NMR (400.1 MHz, C$_6$D$_6$, 300 K): δ** 7.18 (d, 2H, C*H*, Ar*), 7.12 (m, 4H, C*H*$_2$, Ar*), 6.97 (broad m, 15H, C*H*, Ph), 5.03 (s, 1H, C*H*, γ-CH), 3.53 (pent, 4H, C*H*, $^i$Pr), 1.91 (s, 6H, C*H*$_3$, Me), 1.26 (d, 12H, C*H*$_3$, $^i$Pr), 1.01 (d, 12H, C*H*$_3$, $^i$Pr) ppm.

**$^{13}$C{$^1$H} NMR (100.6 MHz, C$_6$D$_6$, 300 K): δ** 163.4 (*C*-CH$_3$, NacNac), 141.4 (*C*H, Ar*), 132.3 (*C*H, Ar, Ph$_3$PO), 132.0 (*C*H, Ar, Ph$_3$PO), 128.9 (*C*H, Ar, Ph$_3$PO), 125.9 (*C*H, Ar, Ph$_3$PO), 123.6 (C$_{quaternary,}$ Ar*), 123.2 (C$_{quaternary}$, Ar*), 122.5 (C$_{quaternary}$, Ar*), 92.9 (*C*H, NacNac), 28.2 (*C*H, $^i$Pr), 24.7 (*C*H, $^i$Pr), 23.6 (*C*H, $^i$Pr), 14.3 (*C*H$_3$, NacNac) ppm.

**$^7$Li NMR (155.5 MHz, C$_6$D$_6$, 300 K): δ** 2.70 (s), 1.52 (broad s, minor unidentified impurity) ppm.

**$^{31}$P{$^1$H} NMR (104.2 MHz, C$_6$D$_6$, 300K): δ** 30.8 ppm.

**IR spectrum:** Analysis run in Nujol mull using solid state FT-IR **γ** ~1738 (imine C=N stretching) cm$^{-1}$.

**Elemental analysis:** Due to the air- and moisture-sensitivity of this compound accurate elemental analysis could not be obtained.

**Melting point analysis**: Compound decomposed to a gel at ~64 °C and became a liquid at ~122 °C.

## Figure S2: ORTEP diagram of [{(MeCN-2,6-$^{i}$Pr$_2$C$_6$H$_3$)$_2$CH}Li.OP(Ph)$_3$] (2)

Hydrogen atoms omitted for clarity. Aryl part of DippNacNac ligand shown as wireframe for clarity.

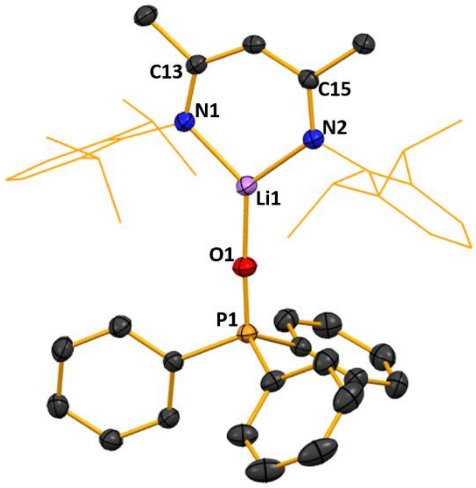


**Selected bond lengths (Å) and bond angles (°)**

Li1 O1 1.810(3), Li1 N1 1.924(3), Li1 N2 1.911(3), P1 O1 1.4886(13), N1 C13 1.318(2), C13 C14 1.407(2), C14 C15 1.412(2), C15 N2 1.317(2).

O1 Li1 N1 131.42(17), O1 Li1 N2 128.79(16), N1 Li1 N2 99.78(15), Li1 O1 P1 176.49(13).

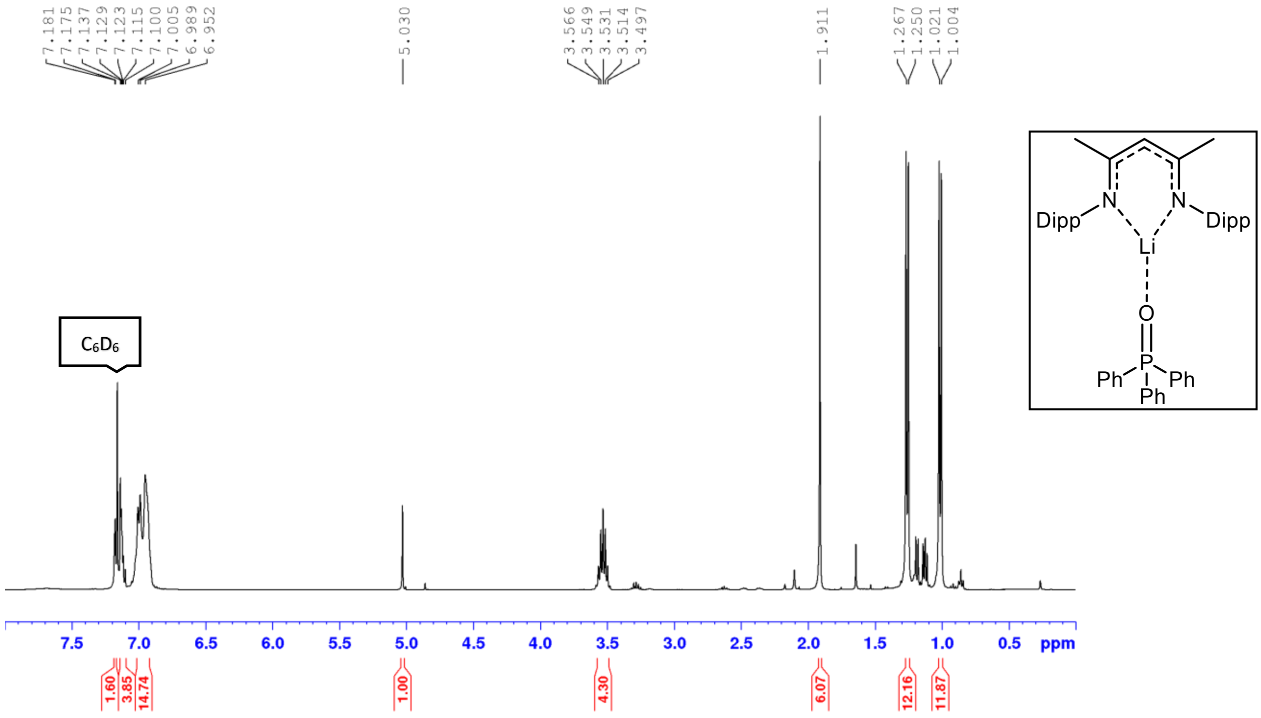


**Figure S2.1**: $^1$H NMR spectrum in C$_6$D$_6$ of **2** [{(MeCN-2,6-iPr$_2$C$_6$H$_3$)$_2$CH}Li.OP(Ph)$_3$]

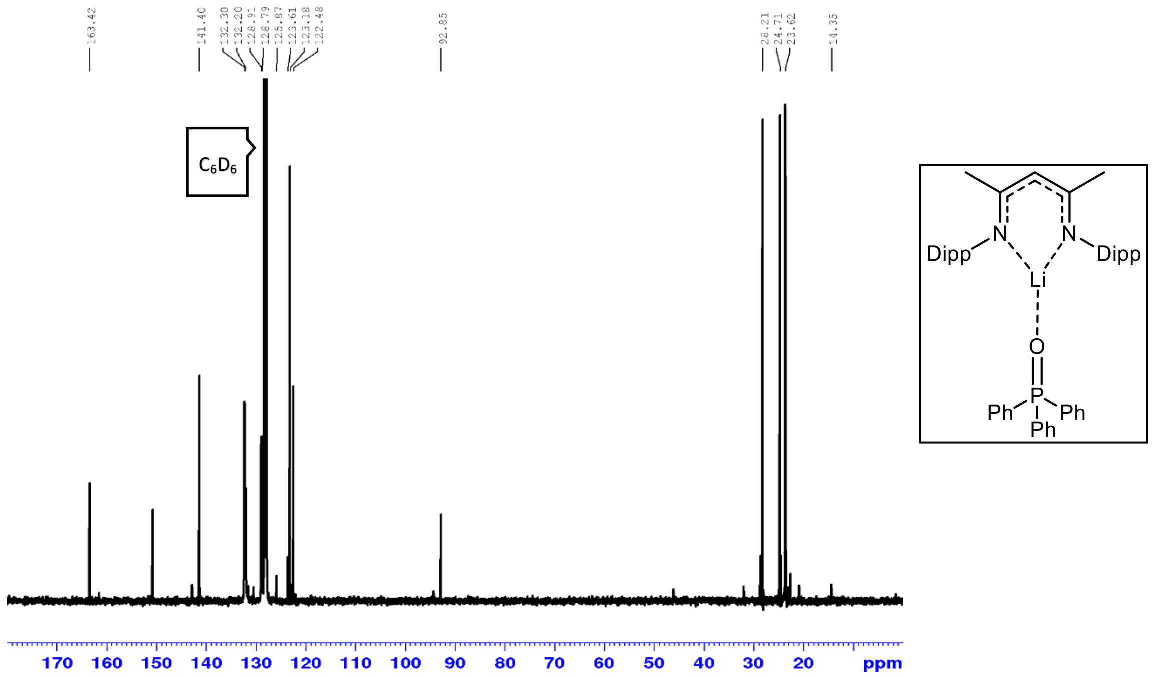


**Figure S2.2**: $^{13}C\{^{1}H\}$ NMR spectrum in $C_6D_6$ of **2** [{(MeCN-2,6-iPr$_2$C$_6$H$_3$)$_2$CH}Li.OP(Ph)$_3$]

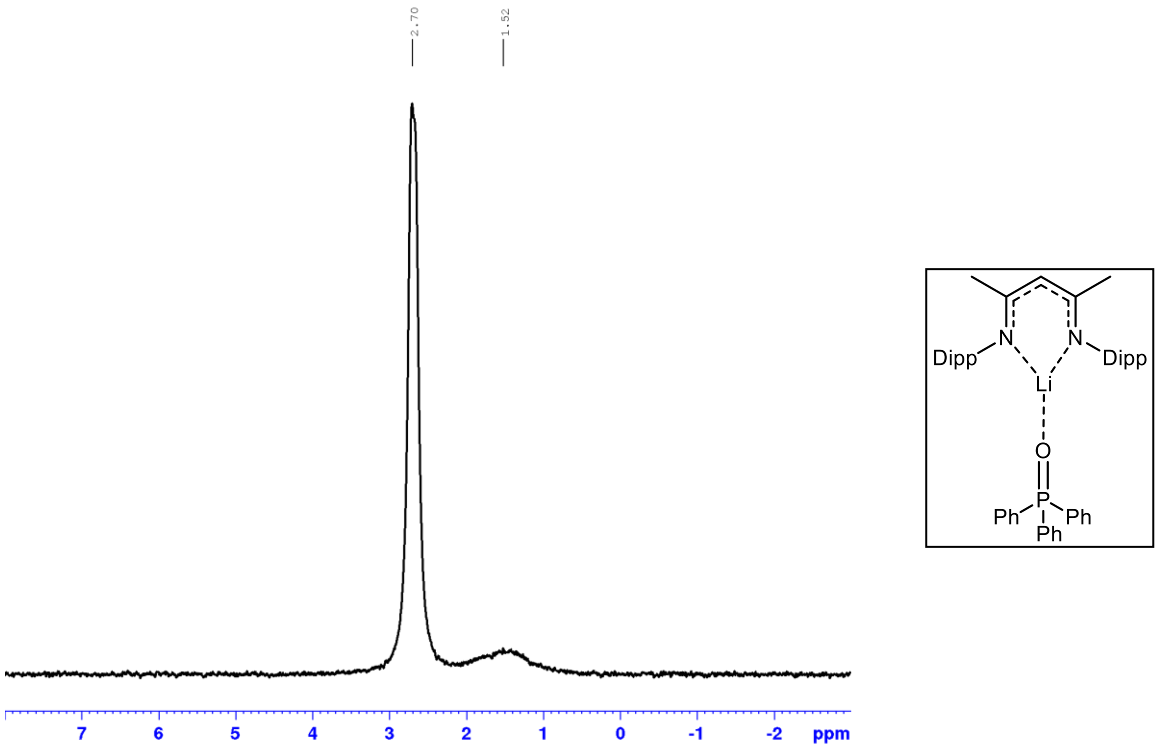


**Figure S2.3**: $^{7}Li$ NMR in $C_6D_6$ of spectrum **2** [{(MeCN-2,6-iPr$_2$C$_6$H$_3$)$_2$CH}Li.OP(Ph)$_3$]

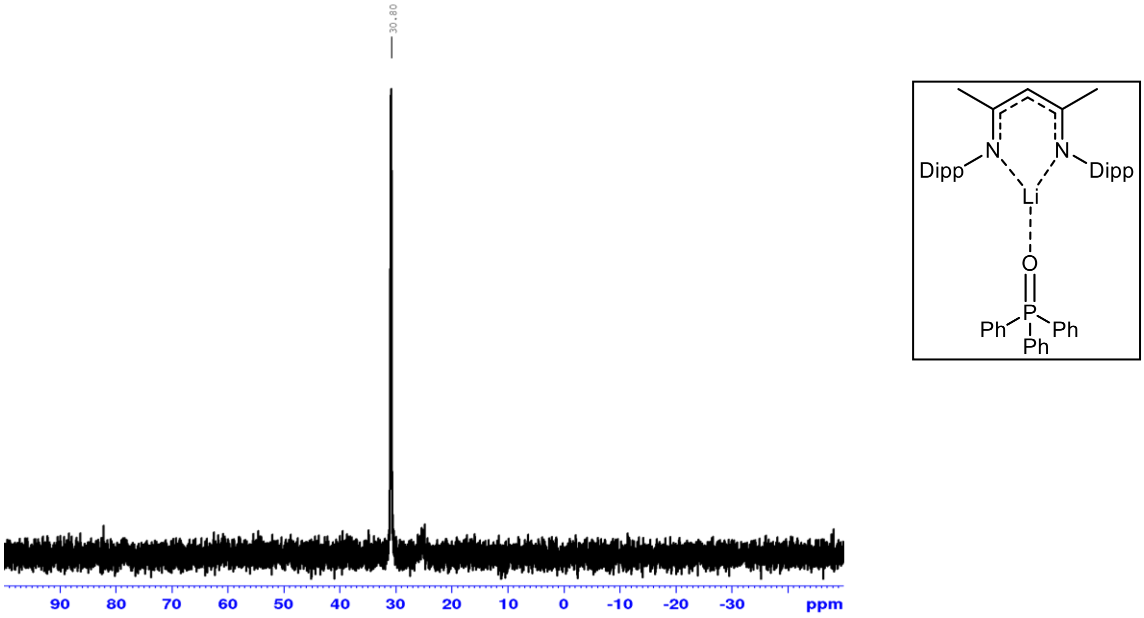


**Figure S2.4**: $^{31}P\{^{1}H\}$ NMR spectrum in $C_6D_6$ of **2** [{(MeCN-2,6-iPr$_2$C$_6$H$_3$)$_2$CH}Li.OP(Ph)$_3$]

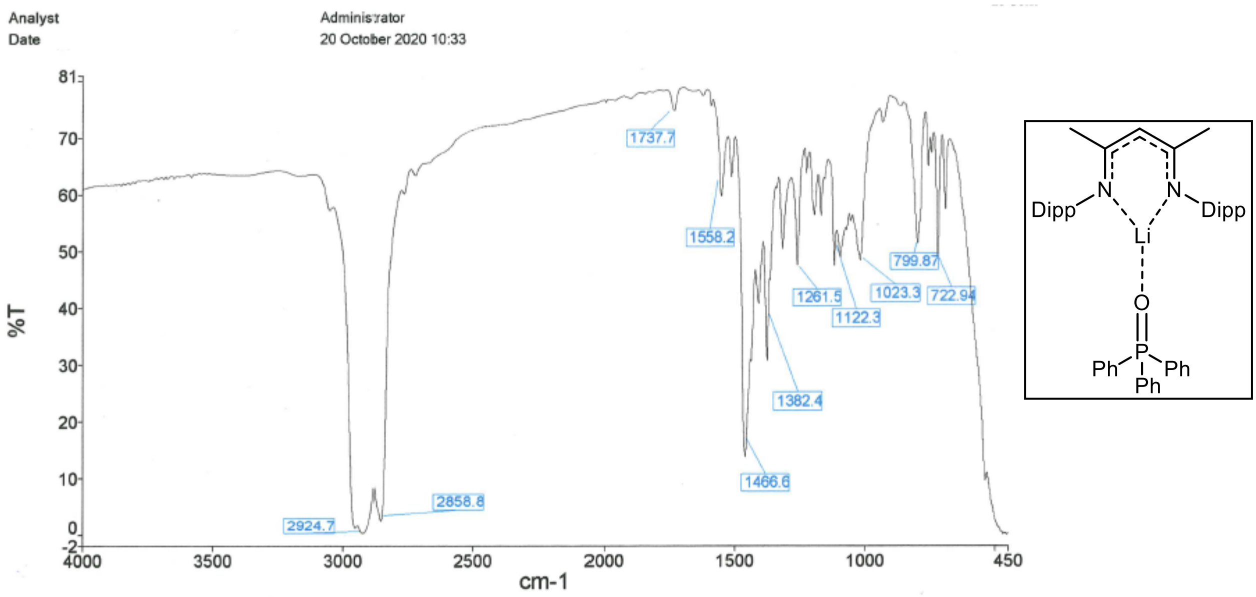


**Figure S2.5**: FT-IR spectrum of **2** [{(MeCN-2,6-iPr$_2$C$_6$H$_3$)$_2$CH}Li.OP(Ph)$_3$]

## Synthesis of [{(MeCN-2,6-$^i$Pr$_2$C$_6$H$_3$)$_2$CH}Li.OC(Ph)$_2$] (**3**)

NacNac(Li) (0.1 mmol, 0.044 g) was added to a glass vial inside the glovebox and dissolved in hexane (1 ml) to which $Ph_2CO$ (0.125 mmol, 0.023 g) was added. Reaction was stirred for 20 minutes prior to addition of an excess of PMDETA to give a red solution. Next the solution was cooled to -20 °C, this produced formed small red crystals within 24 hours (yield 0.036 g, 0.59 mmol, 59 %).

**$^1$H NMR (400.1 MHz, C$_6$D$_6$, 300 K): δ** 7.23 (broad m, 7H, C*H*, Ar*), 6.92 (broad m, 10H, C*H*, Ph), 5.09 (s, 1H, C*H*, γ-CH), 3.54 (sept, 4H, C*H*, $^i$Pr), 1.96 (s, 6H, C$H_3$, Me), 1.28 (d, 12H, C$H_3$, $^i$Pr), 1.01 (d, 12H, C$H_3$, $^i$Pr) ppm.

**$^{13}$C{$^1$H} NMR (100.6 MHz, C$_6$D$_6$, 300 K): δ** 201.0 (C$_{quaternary}$, Ph$_2$*C*O), 163.7 (*C*-CH$_3$, NacNac), 150.2 (C$_{quaternary}$, *C*-NacNac) , 141.4 (C$_{quaternary}$, *Ph*$_2$CO), 136.5 (C$_{quaternary}$, Ar*, $^i$Pr), 133.3 (C$_{quaternary}$, Ar*, $^i$Pr), 131.3 (*C*H, Ar*, *Ph*$_2$CO), 128.7 (*C*H, Ar*, Dipp), 123.5 (*C*H, Ar*, *Ph*$_2$CO), 122.8 (*C*H, Ar*, Dipp), 122.0 (*C*H, Ar*, *Ph*$_2$CO), 93.2 (*C*H, NacNac), 28.3 (*C*H, $^i$Pr), 24.5 (*C*H$_3$, C-Me), 23.4 (*C*H$_3$, $^i$Pr), 22.6 (*C*H$_3$, $^i$Pr) ppm.

**$^7$Li NMR (155.5 MHz, C$_6$D$_6$, 300 K): δ** 3.64 (s), 1.20 (broad s, minor unidentified impurity) ppm.

**IR spectrum:** Analysis run in Nujol mull using solid state FT-IR **γ** ~1738 (imine C=N stretching) cm$^{-1}$.

**Elemental analysis:** Due to the air- and moisture-sensitivity of this compound accurate elemental analysis could not be obtained.

**Melting point analysis**: Compound decomposed to a gel at ~68 °C and became a liquid at ~96 °C.

## Figure S3: ORTEP diagram of [{(MeCN-2,6-iPr$_2$C$_6$H$_3$)$_2$CH}Li.OC(Ph)$_2$] (**3**)

Hydrogen atoms omitted for clarity. Aryl part of DippNacNac ligand shown as wireframe for clarity.

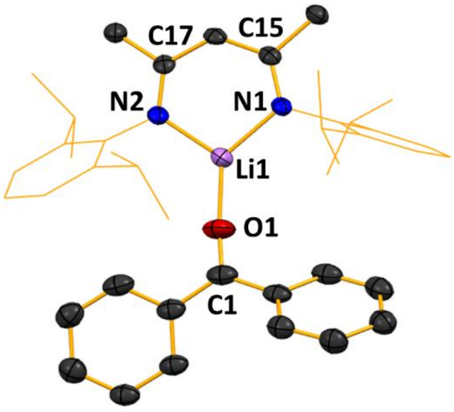


**Selected bond lengths (Å) and bond angles (°)**

Li1 O1 1.843(3), Li1 N1 1.907(3), Li1 N2 1.914(3), C1 O1 1.232(3), N1 C15 1.326(2), C15 C16 1.410(2), C16 C17 1.408(2), C17 N2 1.318(2).

O1 Li1 N1 133.93(18), O1 Li1 N2 124.80(17), N1 Li1 N2 101.25(14), Li1 O1 C1 170.0(2).

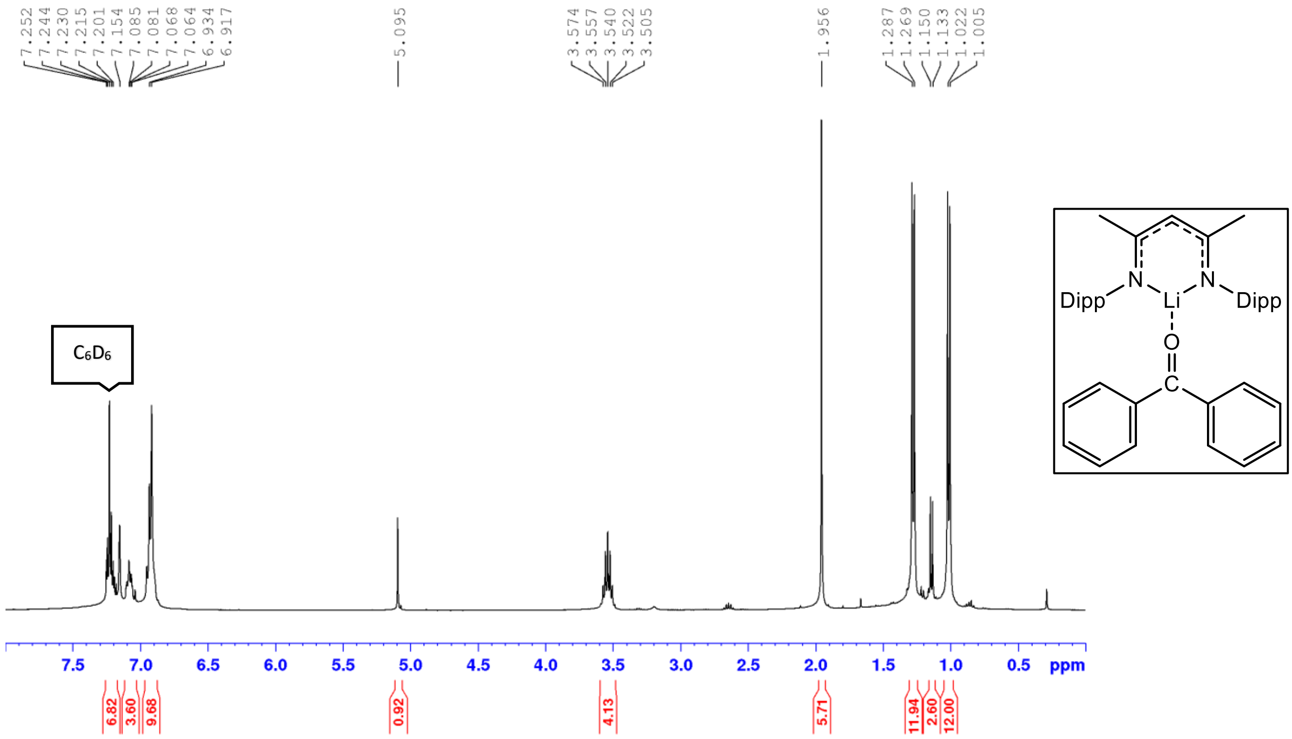


**Figure S3.1**: $^1$H NMR spectrum in $C_6D_6$ of **3** [{(MeCN-2,6-iPr$_2$C$_6$H$_3$)$_2$CH}Li.OC(Ph)$_2$]

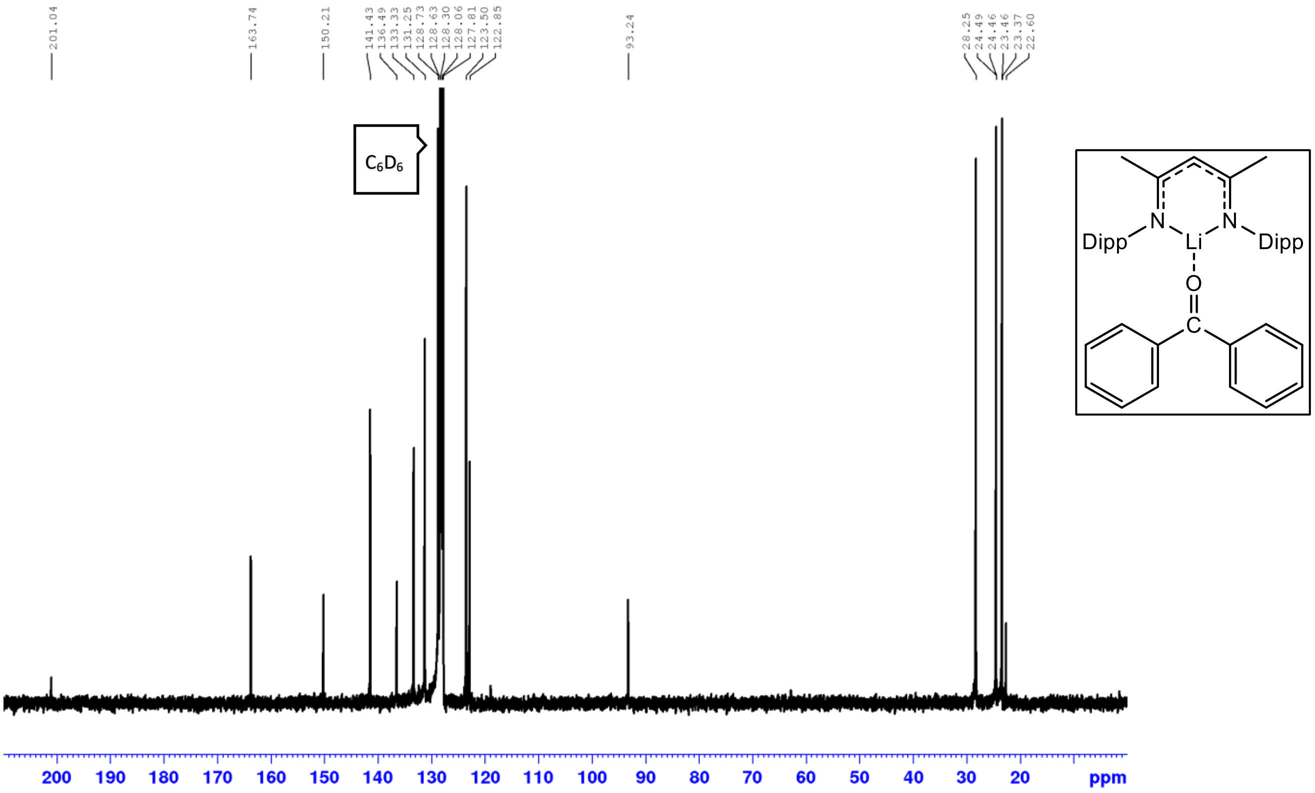


**Figure S3.2**: $^{13}C\{^{1}H\}$ NMR in $C_6D_6$ of spectrum **3** [{(MeCN-2,6-iPr$_2$C$_6$H$_3$)$_2$CH}Li.OC(Ph)$_2$]

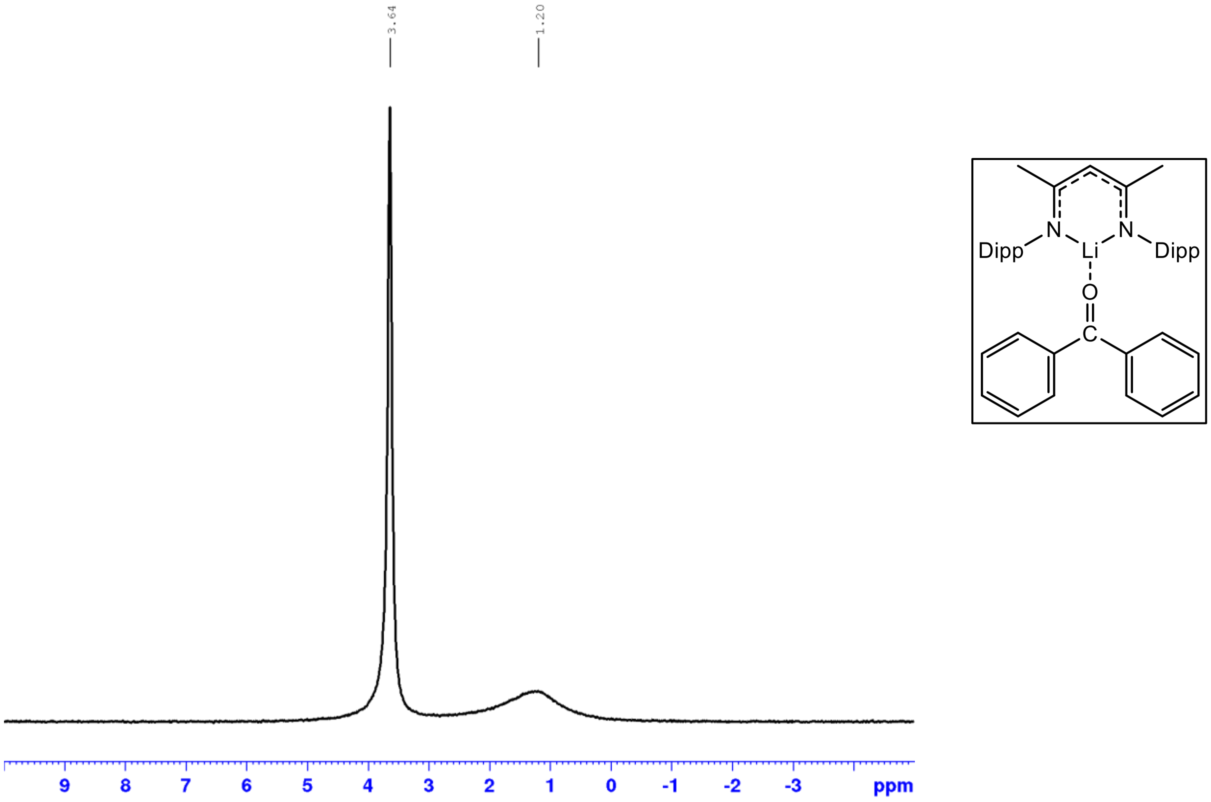


**Figure S3.3**: $^{7}Li$ NMR in $C_6D_6$ of spectrum **3** [{(MeCN-2,6-iPr$_2$C$_6$H$_3$)$_2$CH}Li.OC(Ph$_2$]

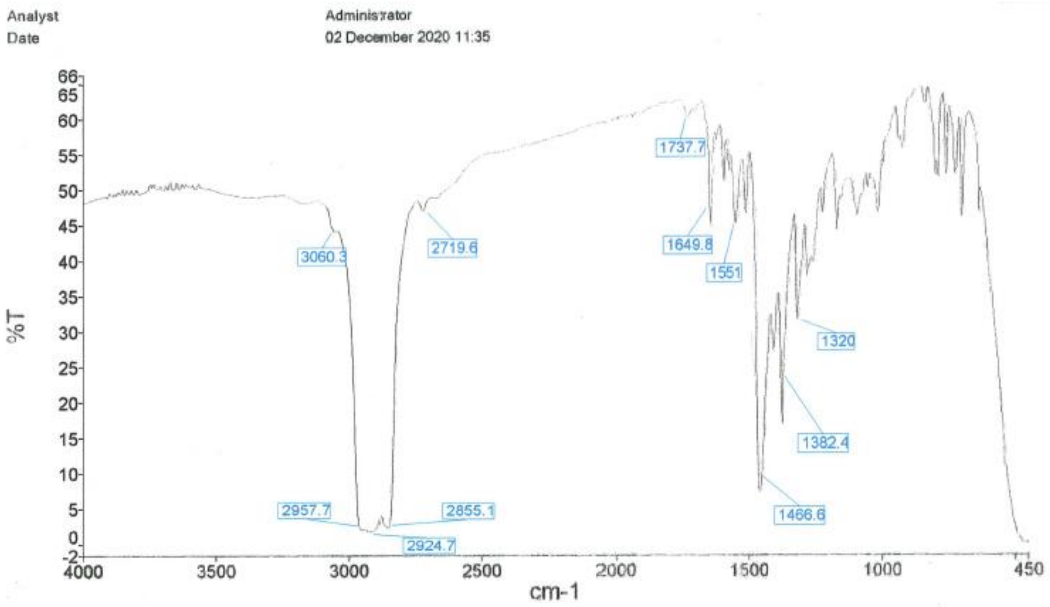


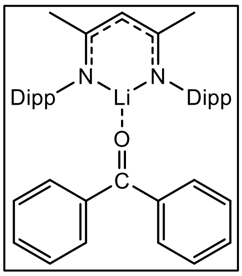


**Figure S3.4**: FT-IR spectrum of **3** [{(MeCN-2,6-iPr$_2$C$_6$H$_3$)$_2$CH}Li.OC(Ph)$_2$]

## Synthesis of [{(MeCN-2,6-$^{i}$Pr$_2$C$_6$H$_3$)$_2$CH(CON($^{t}$Bu)}Mg(CON($^{t}$Bu))TMP] (**4**)

[{(MeCN-2,6-$^{i}$Pr$_2$C$_6$H$_3$)$_2$CH}Mg(TMP)] (0.5 mmol, 0.291 g) was placed in a Schlenk flask and dissolved in hexane (5 ml) before $^{t}$BuNCO (0.5 mmol, 60 µl) was added at which point a clear yellow solution had formed. The solution was left stirring for 30 mins whereupon a cloudy suspension was produced. This suspension was concentrated *in vacuo* before being gently heated while THF solvent (9.2 mmol, 0.75 ml) was introduced slowly by syringe. Rapid formation of pale-yellow crystals then occurred (yield 0.102 g, 0.13 mmol, 26 %).

**$^{1}$H NMR (400.1 MHz, C$_6$D$_6$, 300 K): δ** 7.09 (broad m, 6H, C*H*, Ar*), 4.80 (s, 1H, C*H*, γ-CH), 3.08 (m, 2H, C*H*, $^{i}$Pr), 2.95 (m, 2H, C*H*, $^{i}$Pr), 1.75 (s, 9H, C*H*$_3$, $^{t}$Bu), 1.58 (s, 6H, C*H*$_2$, TMP), 1.47 (s, 12H, C*H*$_3$, TMP), 1.34 (d, 12H, C*H*$_3$, $^{i}$Pr), 1.19 (d, 12H, C*H*$_3$, $^{i}$Pr), 1.14 (s, 6H, C*H*$_3$, Me) ppm.

**$^{13}$C{$^{1}$H} NMR (100.6 MHz, C$_6$D$_6$, 300 K): δ** 176.3 (C$_{quaternary}$, N*C*O), 142.0 (C$_{quaternary}$, *C*-Me), 139.2 (C$_{quaternary}$, Ph), 138.6 (C$_{quaternary}$, $^{i}$Pr), 125.6 (*C*H, Ph), 123.5 (*C*H, Ph), 122.9 (*C*H, Ph), 68.8 (C$_{quaternary}$, $^{t}$Bu), 51.2 (C$_{quaternary}$, $^{t}$Bu), 39.9 (C$_{quaternary}$, TMP), 36.3 (*C*H, TMP), 32.5 (C$_{quaternary}$, $^{i}$Pr), 29.8 (C$_{quaternary}$, $^{i}$Pr), 29.5 (*C*H$_3$, $^{t}$Bu), 29.1 (*C*H$_3$, $^{t}$Bu), 28.9 (*C*H$_3$, $^{t}$Bu), 28.1 (*C*H$_3$, $^{t}$Bu), 27.6 (*C*H$_3$, Me, TMP), 27.5 (*C*H$_3$, Me, TMP), 26.7 (*C*H$_3$, $^{i}$Pr), 24.7 (*C*H$_3$, $^{i}$Pr), 24.4 (*C*H$_3$, $^{i}$Pr), 24.0 (*C*H$_3$, $^{i}$Pr), 23.8 (*C*H$_3$, $^{i}$Pr) , 23.3 (*C*H$_3$, $^{i}$Pr), 17.7 (*C*H$_3$, NacNac) ppm.

**IR:** Analysis run in Nujol mull using solid state FT-IR **γ** ~3000 (aromatic CH stretching), 1734 (C=O), ~1500 (aliphatic CH bending) cm$^{-1}$.

**Elemental analysis:** Due to the air- and moisture-sensitivity of this compound accurate elemental analysis could not be obtained.

**Melting point analysis**: Compound decomposed to a gel at ~112 °C and became a liquid at ~164 °C.

## Figure S4: ORTEP diagram of [{(MeCN-2,6-$^{i}Pr_2C_6H_3$)$_2$CH(CON($^{t}$Bu)}Mg(CON($^{t}$Bu))TMP] (**4**)

Hydrogen atoms omitted for clarity. Aryl part of DippNacNac ligand shown as wireframe for clarity.

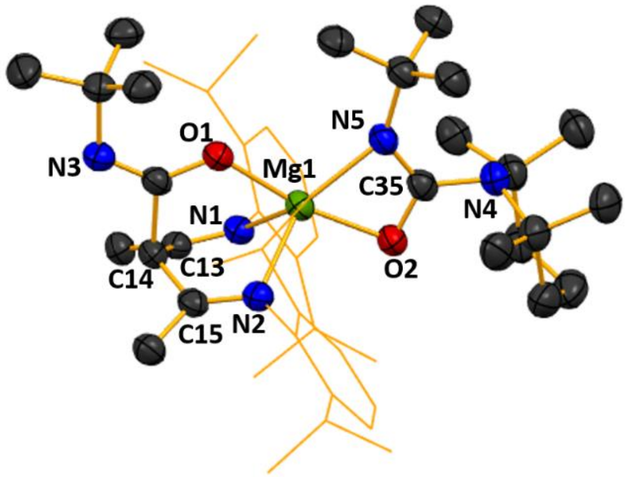


**Selected bond lengths (Å) and bond angles (°)**

Mg1 O1 1.986(2), Mg1 N1 2.162(3), Mg1 N2 2.163(3), Mg1 O2 2.020(2), Mg1 N5 2.106(3), C13 N1 1.279(4), C13 C14 1.513(4), C14 C15 1.520(4), C15 N2 1.279(4), C35 N5 1.301(4), C35 O2 1.292(4), C35 N4 1.434(4).

O1 Mg1 N1 86.92(9), O1 Mg1 N2 86.68(9), N1 Mg1 N2 88.21(10), N5 Mg1 O2 64.49(9).

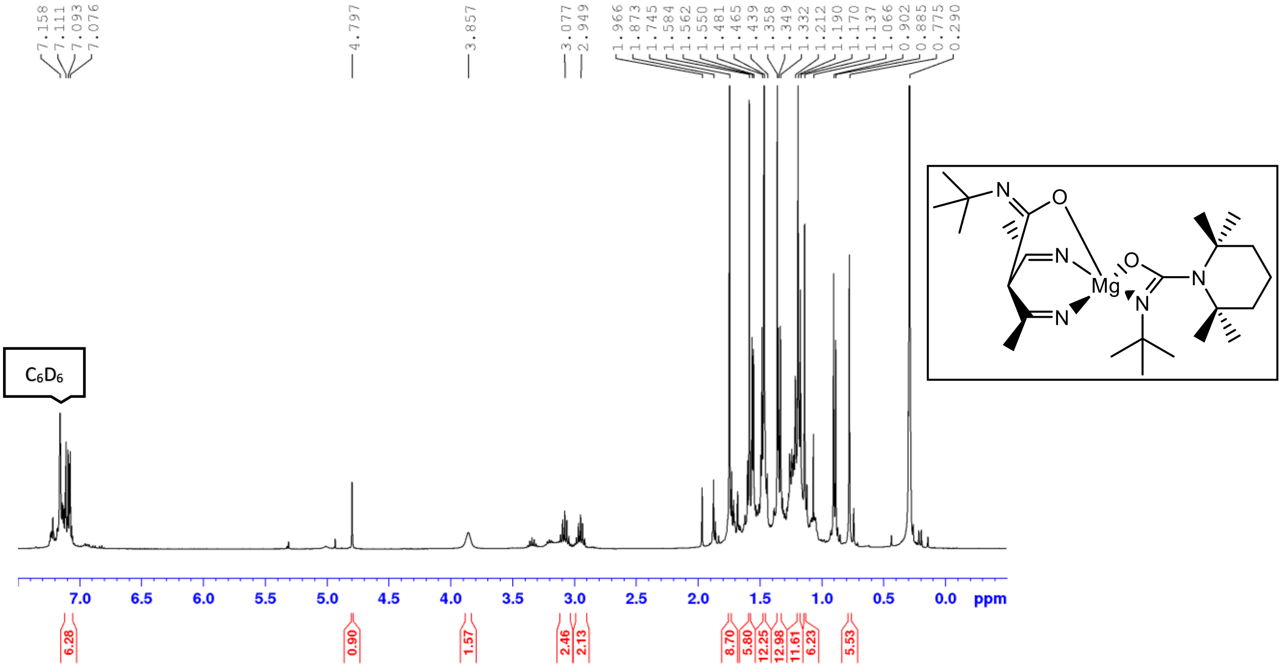


**Figure S4.1**: $^{1}$H NMR spectrum in $C_6D_6$ of compound **4** [{(MeCN-2,6-$^{i}Pr_2C_6H_3$)$_2$CH(CON($^{t}$Bu)}Mg(CON($^{t}$Bu))TMP]

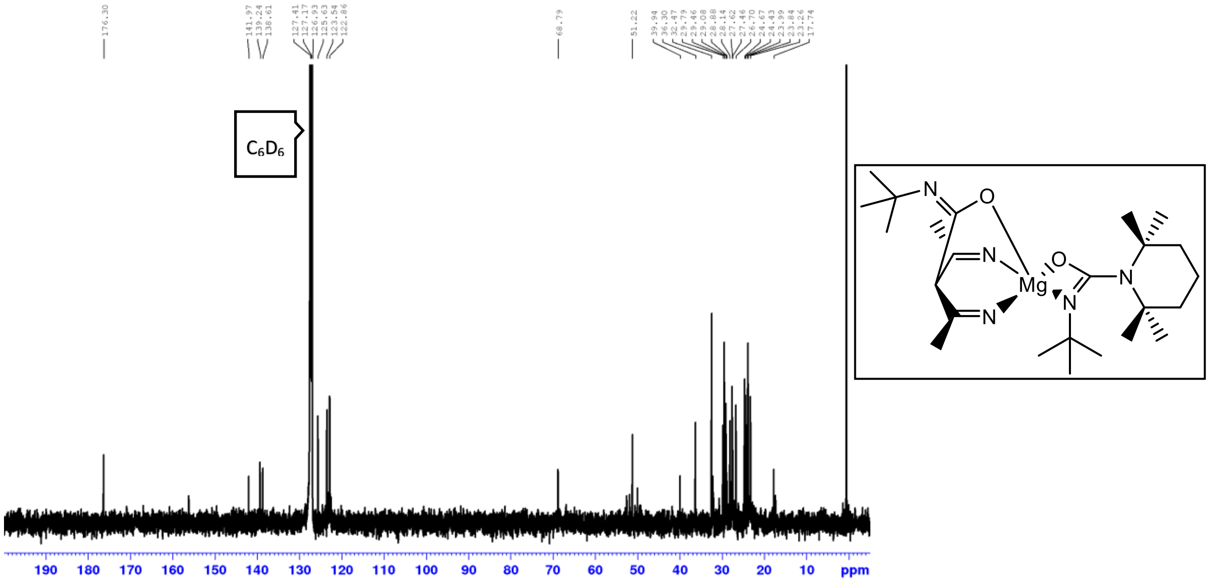


**Figure S4.2**: $^{13}C\{^1H\}$ NMR spectrum in $C_6D_6$ of compound **4** [{(MeCN-2,6-$^iPr_2C_6H_3)_2CH(CON(^tBu)\}Mg(CON(^tBu))TMP]

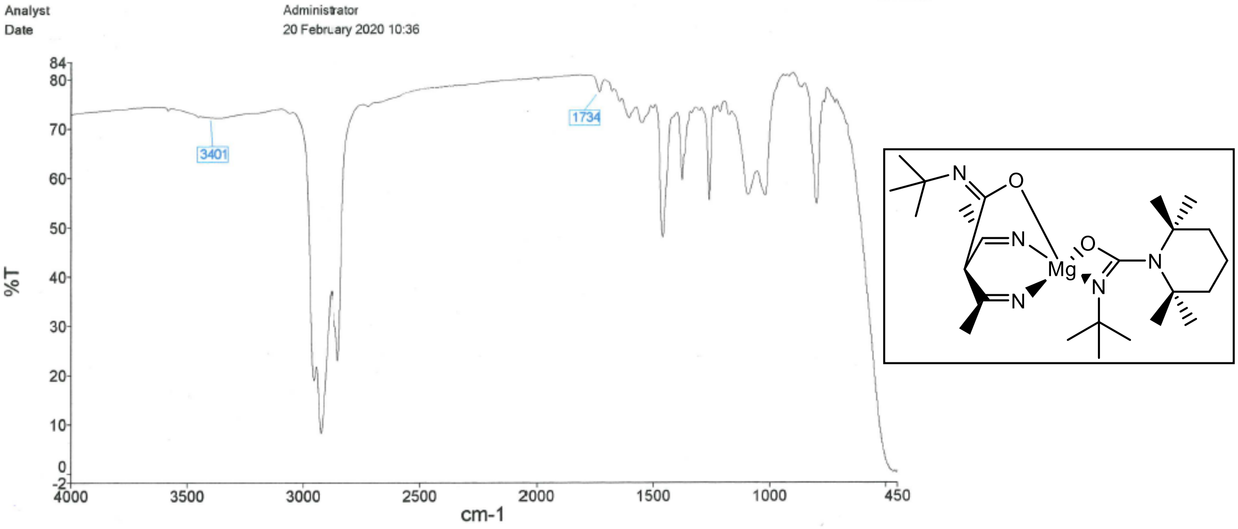


**Figure S4.3**: FT-IR spectrum of compound **4** [{(MeCN-2,6-$^iPr_2C_6H_3)_2CH(CON(^tBu)\}Mg(CON(^tBu))TMP]

## Synthesis of [{(MeCN-2,6-$^i$Pr)$_2$C$_6$H$_3$)$_2$CH}Mg(TMP)($^t$BuNCS)] (5)

[{(MeCN-2,6-$^i$Pr$_2$C$_6$H$_3$)$_2$CH}Mg(TMP)] (0.5 mmol, 0.290 g) was placed in Schlenk flask and dissolved in hexane solvent (5 ml) before $^t$BuNCS (0.5 mmol, 60 µl) was added, to produce a transparent orange solution. Left stirring for 30 mins, this solution slowly darkened from a clear orange to a clear brown solution. The solution was then concentrated *in vacuo*, at which point solid began to precipitate, so gentle heating was applied together with the slow addition of THF solvent (12.3 mmol, 1 ml) until a clear brown solution was once again obtained and the Schlenk flask surrounded by a warm bath. A small batch of clear colorless crystals formed overnight (yield 0.286 g, 0.4 mmol, 83 %).

**$^1$H NMR (400.1 MHz, C$_6$D$_6$, 300 K): δ** 7.12 (broad m, 4H, C*H*, Ar*), 7.08 (broad m, 2H, C*H*, Ar*), 4.78 (s, 1H, C*H*$_2$, γ-CH), 3.33 (m, 4H, C*H*, $^i$Pr), 1.85 (s, 9H, C*H*$_3$, $^t$Bu), 1.53 (s, 6H, C*H*$_2$, TMP), 1.32 (s, 6H, C*H*$_3$, Me), 1.27 (s, 3H, C*H*$_3$, TMP), 1.26 (s, 3H, C*H*$_3$, TMP), 1.20 (d, 24H, C*H*$_3$, $^i$Pr), 1.13 (s, 3H, C*H*$_3$, TMP), 1.12 (s, 3H, C*H*$_3$, TMP) ppm.

**$^{13}$C{$^1$H} NMR (100.6 MHz, C$_6$D$_6$, 300 K): δ** 169.5 (C$_{quaternary}$, N*C*S), 146.9 (C$_{quaternary}$, *C*-Me, NacNac), 142.5 (C$_{quaternary}$, Ph), 141.5 (C$_{quaternary}$, $^i$Pr), 125.4 (*C*H, Ar*),123.8 (*C*H, Ar*), 123.4 (*C*H, Ar*), 95.1 (C$_{quaternary}$, TMP), 55.9 (C$_{quaternary}$, $^t$Bu), 53.5 (*C*H$_2$, NacNac), 37.7 (*C*H$_2$, TMP), 33.6 (*C*H, $^i$Pr), 31.2 (*C*H, $^i$Pr), 28.5 (*C*H, $^i$Pr), 28.4 (*C*H, $^i$Pr), 27.8 (*C*H$_3$, TMP), 26.0 (*C*H$_3$, TMP), 24.9 (*C*H, $^i$Pr), 23.9 (*C*H, $^i$Pr), 23.4 (*C*H, $^i$Pr), 23.1 (*C*H, $^i$Pr), 16.7 (*C*H$_3$, NacNac) ppm.

**IR:** Analysis run in Nujol mull using solid state FT-IR **γ** ~2148 (carbodiimide N=C=N stretching), ~1734 (imine C=N stretching) cm$^{-1}$.

**Elemental analysis:** Due to the air- and moisture-sensitivity of this compound accurate elemental analysis could not be obtained.

**Melting point analysis**: Compound became a liquid in the range 126-128°C.

## Figure S5: ORTEP diagram of [{(MeCN-2,6-$^{i}$Pr)$_2$C$_6$H$_3$)$_2$CH}Mg(TMP)($^{t}$BuNCS)] (5)

There are two crystallographically independent molecules featured in the structure, though only one is presented. Hydrogen atoms omitted for clarity. Aryl part of DippNacNac ligand shown as wireframe for clarity.

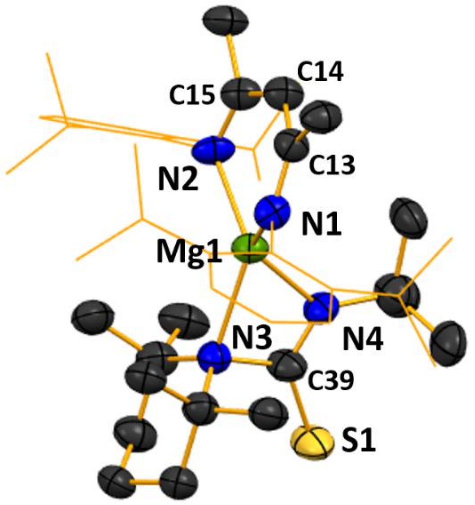


**Selected bond lengths (Å) and bond angles (°)**

Mg1 N1 2.100(6), Mg1 N2 2.103(5), Mg1 N3 2.164(6), Mg1 N4 2.045(6), C13 N1 1.329(9), C13 C14 1.411(9), C14 C15 1.398(9), C15 N2 1.327(9), C39 N3 1.506(9), C39 N4 1.294(10), C35 S1 1.701(7).

N1 Mg1 N2 94.85(2), N3 Mg1 N4 63.32(2), N3 C39 S1 121.97(6), N4 C39 S1 130.04(6).

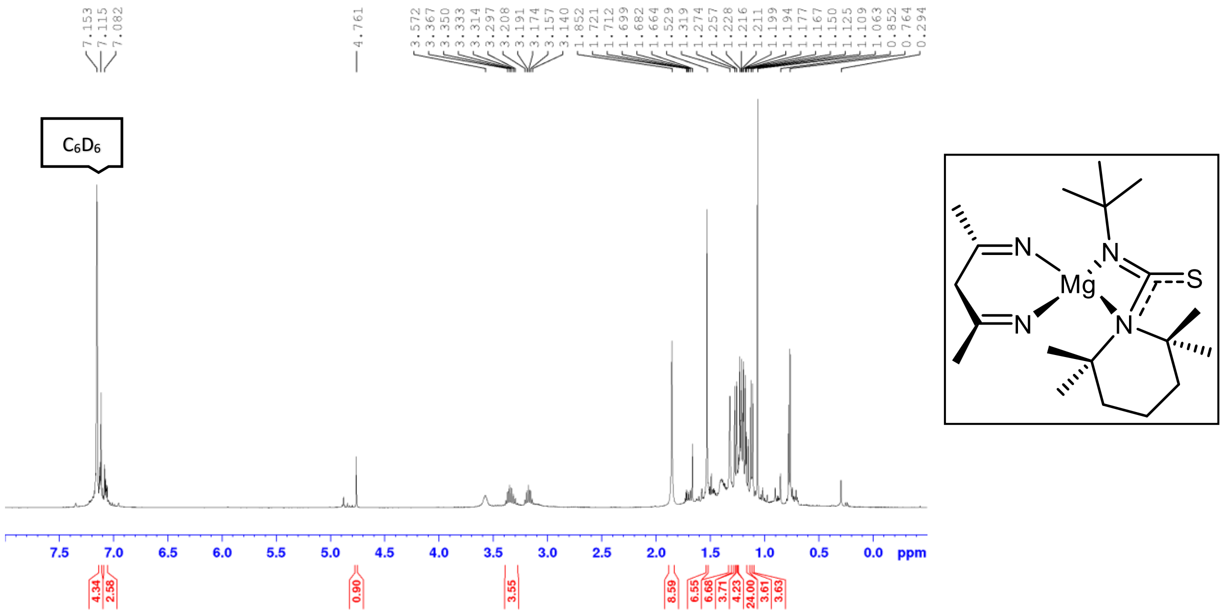


**Figure S5.1**: $^{1}$H NMR spectrum in C$_6$D$_6$ of **5** [{(MeCN-2,6-$^{i}$Pr)$_2$C$_6$H$_3$)$_2$CH}Mg(TMP)($^{t}$BuNCS)]

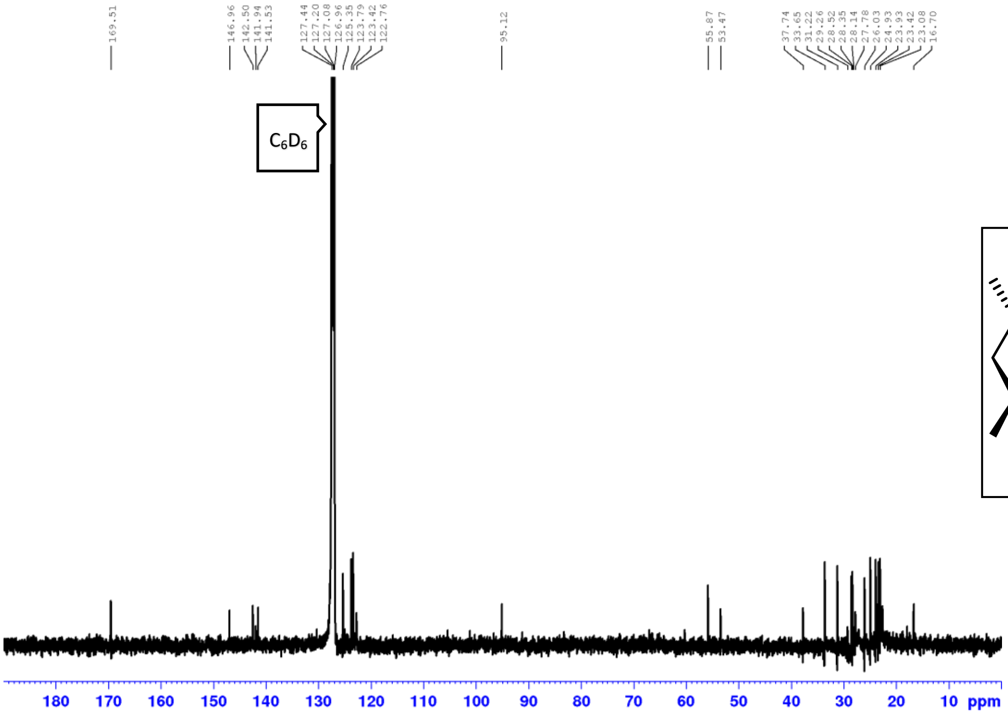


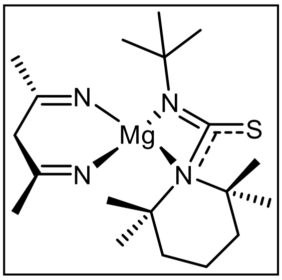


**Figure S5.2**: $^{13}C\{^{1}H\}$ NMR spectrum in $C_6D_6$ of compound **5** [{(MeCN-2,6-$^{i}Pr)_2C_6H_3)_2CH\}Mg(TMP)(^{t}BuNCS)$]

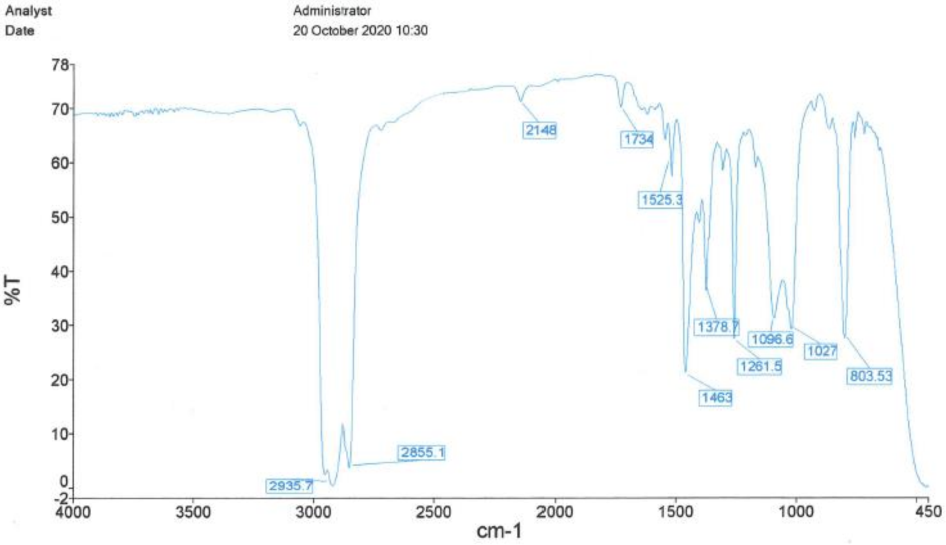


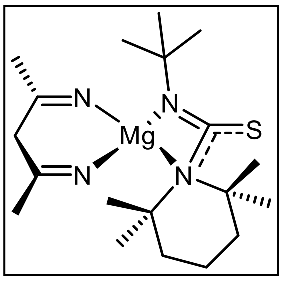


**Figure S5.3**: FT-IR spectrum of compound **5** [{(MeCN-2,6-$^{i}Pr)_2C_6H_3)_2CH\}Mg(TMP)(^{t}BuNCS)$]

## Table S1: Selected X-ray crystal structural data and refinement details for compounds 1-5

| **Compound** | **[{(MeCN-2,6-$^{i}$Pr2C6H3)2CH}Li.N(Cy)CN(Cy)] 1** | **[{(MeCN-2,6-$^{i}Pr_2C_6H_3)_2$CH}Li.OP(Ph)$_3$] 2** | **[{(MeCN-2,6-$^{i}Pr_2C_6H_3)_2$CH}Li.OC(Ph)$_2$] 3** | **[{(MeCN-2,6-$^{i}Pr_2C_6H_3)_2$CH(CON(*t*-Bu)}Mg(CON(*t*-Bu))TMP] 4** | **[{(MeCN-2,6-$^{i}Pr)_2C_6H_3)_2$CH}Mg(TMP)(t-BuNCS)] 5** |
|---|---|---|---|---|---|
| **Empirical Formula** | $C_{42}H_{63}LiN_4$*<br>*Not inc. disordered solvent removed with SQUEEZE | $C_{100}H_{126}Li_2N_4O_2P_2$ | $C_{42}H_{51}LiN_2O$ | $C_{60}H_{105}MgN_5O_2$ | $C_{86}H_{136}Mg_2N_8S_2$ |
| **Molecular Mass** | 630.90 | 1491.86 | 606.78 | 952.79 | 1394.76 |
| **Λ** | Cu Kσ (λ = 1.54184) | Cu Kσ (λ = 1.54184) | Cu Kσ (λ = 1.54184) | Cu Kσ (λ = 1.54184) | Cu Kσ (λ = 1.54184) |
| **Space Group** | P-1 | C2/c | P2$_1$/c | P-1 | Pna2$_1$ |
| **Crystal system** | Triclinic | Monoclinic | Monoclinic | Triclinic | Orthorhombic |
| **Temperature (K)** | 123(2) | 123(2) | 123(2) | 123(2) | 123(2) |
| **a/Å** | 12.4391(5) | 20.0377(3) | 14.0637(2) | 12.2658(7) | 36.608(2) |
| **b/Å** | 14.1774(7) | 16.6030(3) | 15.7355(2) | 16.4965(9) | 20.2809(9) |
| **c/Å** | 24.4260(8) | 27.2162(3) | 16.8566(2) | 17.2191(10) | 11.2327(5) |
| **α/°** | 92.321(3) | 90 | 90 | 73.351(5) | 90 |
| **β/°** | 102.549(3) | 92.942(1) | 99.962(1) | 69.258(5) | 90 |
| **γ/°** | 97.521(4) | 90 | 90 | 70.059(5) | 90 |
| **Volume/Å$^3$** | 4158.0(3) | 9042.5(2) | 3674.11(8) | 3008.1(3) | 8339.6(7) |
| **Z** | 4 | 4 | 4 | 2 | 4 |
| **Measured Reflections** | 34426 | 22030 | 22556 | 20753 | 39211 |
| **Unique Reflections** | 15647 | 8878 | 7236 | 20753 | 13472 |
| **$R_{int}$** | 0.0725 | 0.0310 | 0.0726 | 0.0809<br>Before twin treatment | 0.0915 |

| | | | | | |
|---|---|---|---|---|---|
| **Observed Reflections [I>2σI]** | 8349 | 7056 | 5519 | 10893 | 9279 |
| **Goodness of Fit** | 0.895 | 1.030 | 1.041 | 0.909 | 1.048 |
| **R [on F, obs refs only]** | 0.0718 | 0.0506 | 0.0563 | 0.0800 | 0.0887 |
| **ωR [on $F^2$, all data]** | 0.2031 | 0.1452 | 0.1712 | 0.2346 | 0.2803 |
| **Largest diff. peak /hole/$Å^{-3}$** | 0.673/-0.323 | 0.423/-0.276 | 0.334/-0.253 | 0.552/-0.265 | 0.774/-0.396 |

# References


1. M. Stender, R. J. Wright, B. E. Eichler, J. Prust, M. M. Olmstead, H. W. Roesky and P. P. Power, *J. Chem. Soc., Dalton Trans.*, 2001, 3465-3469.
2. S. E. Baillie, V. L. Blair, T. D. Bradley, W. Clegg, J. Cowan, R. W. Harrington, A. Hernán-Gómez, A. R. Kennedy, Z. Livingstone and E. Hevia, *Chem. Sci.*, 2013, **4**, 1895-1905.
3. Agilent, CrysalisPrp, Agilent Technologies Ltd., Yarnton, Oxfordshire, England. 2014.
4. G. M. Sheldrick, *Acta Crystallogr.*, 2015, **C71**, 3-8.
5. O. V. Dolomanov, L. J. Bourhis, R. J. Gildea, J. A. K. Howard and H. Puschmann, *J. Appl. Cryst.*, 2009, **42**, 339-341.
6. A. L. Spek, *Acta Crystallogr.*, 2015, **C71**, 9-18.
